# From developmental divergence to degenerative vulnerability: convergent molecular mechanisms linking autism spectrum disorder and Alzheimer’s disease

**DOI:** 10.3389/fnmol.2026.1963972

**Published:** 2026-10-21

**Authors:** Christian Griñán-Ferré, Fahad Hussain, Aina Bellver-Sanchis, Núria Pérez-Salvador, Yeminia Valle, Daniel Ortuño-Sahagún, Oscar Arias-Carrión

**Affiliations:** 1 Department of Pharmacology, Toxicology, and Therapeutic Chemistry, University of Barcelona, Barcelona, Spain; 2 Institute of Neurosciences of the University of Barcelona, University of Barcelona, Barcelona, Spain; 3 Centro de Investigación en Red, Enfermedades Neurodegenerativas (CIBERNED), Instituto de Salud Carlos III, Madrid, Spain; 4 Departamento de Neurociencias, Instituto de Neurociencias Traslacionales, Centro Universitario de Ciencias de la Salud (CUCS), Universidad de Guadalajara, Guadalajara, Jalisco, Mexico; 5 División de Neurociencias Clínica, Instituto Nacional de Rehabilitación Luis Guillermo Ibarra Ibarra, Mexico City, Mexico; 6 Escuela de Medicina y Ciencias de la Salud, Tecnologico de Monterrey, Mexico City, Mexico

**Keywords:** Alzheimer’s disease, attractor dynamics, autism spectrum disorder, heterochrony, neurodegeneration, neurodevelopmental disorders, neurodevelopmental timing, synaptic pruning deficit

## Abstract

Neurodevelopmental disorders (NDDs) and neurodegenerative diseases (NDEs) have long been regarded as temporally and mechanistically distinct: NDDs such as autism spectrum disorder (ASD), Fragile X syndrome, Prader–Willi syndrome, and Phelan–McDermid syndrome arise during critical periods of brain maturation, whereas NDEs such as Alzheimer’s disease (AD) manifest later with progressive neuronal loss, protein aggregation, and cognitive decline. Accumulating genetic, molecular, cellular, and epidemiological data now suggests substantial overlaps, indicating that diverse genetic and environmental insults converge on a limited set of shared biological pathways across the lifespan. Large-scale genomic analyses have identified at least 148 genes shared between ASD and AD, but this overlap should be understood as a shared regulatory architecture rather than evidence of a common causal pathway. About half of these genes are linked to the mTOR signaling pathway, which regulates protein synthesis, autophagy, and synaptic homeostasis. Epidemiological studies indicate an increased occurrence of early-onset dementia among adults with ASD, although available studies do not establish that this association is specific to Alzheimer’s disease or to idiopathic ASD. Both conditions involve dysregulation of synaptic scaffolding (e.g., SHANK3), epigenetic regulation (including G9a/EHMT2-mediated H3K9 methylation), complement-dependent synaptic pruning, mitochondrial and metabolic homeostasis, neuro-inflammation, and glymphatic clearance. We propose a unifying framework, presented as a testable hypothesis rather than an established mechanism, in which NDDs and NDEs represent distinct temporal manifestations within a shared evolutionary trade-off landscape balancing developmental connectivity, metabolic cost, and long-term proteostatic resilience. Within this landscape, convergent nodes—mTOR–autophagy, G9a/EHMT2-mediated chromatin regulation, complement-mediated pruning, and glymphatic function—may contribute to differences in early circuit formation and have been proposed as potential sources of later vulnerability. The APP–FMRP–mGluR5 axis further illustrates molecular convergence between monogenic NDDs and pathways relevant to AD. This review uses ASD and AD as paradigmatic examples to illustrate how heterochronic disruptions in synaptic maturation and proteostasis may create a lifespan continuum from atypical neurodevelopment to accelerated degeneration, while explicitly noting that this framework remains a testable hypothesis and does not establish a causal progression from ASD to AD. Recognizing these shared nodes opens avenues for transdiagnostic biomarkers, longitudinal risk stratification, and multitarget therapies—including epigenetic modulators, mTOR regulators, and glymphatic enhancers—that may benefit both ends of the continuum.

## 1 Introduction

Neurodevelopmental disorders and neurodegenerative diseases have historically occupied separate conceptual and clinical territories. NDDs—including ASD, Fragile X syndrome (FXS), Prader-Willi syndrome (PWS), and Phelan-McDermid syndrome (PMS—manifest early in brain development through disrupted neural circuit formation, abnormal synaptic connectivity, and enduring cognitive and behavioral alterations. NDEs, such as Alzheimer’s disease (AD), Parkinson’s disease, and Huntington’s disease, emerge after decades of apparently normal neural function and are marked by progressive neuronal death, pathological protein aggregation, and irreversible cognitive decline (Lord et al., 2020; Wilson et al., 2023). The apparent simplicity of this temporal dichotomy has long justified treating these conditions as categorically distinct.

The conceptual shift toward a shared vulnerability continuum has been driven by several converging lines of evidence. First, large-scale genomic studies have shown that many NDD risk genes encode proteins that function not only during development but also across the lifespan, supporting synaptic plasticity and proteostasis in the adult brain (Satterstrom et al., 2020). Second, as medical advances have extended the survival of individuals with genetic NDDs into middle age, progressive neurological decline resembling neurodegenerative processes has been increasingly documented (Manzo and Hernández-Aguilar, 2026). Third, the literature has identified shared disruptions across pathways, including synaptic architecture, epigenetic regulation, mitochondrial metabolism, and neuro-inflammation, that span the traditional developmental-degenerative boundary (Liao et al., 2026; Manzo and Hernández-Aguilar, 2026).

An innovative pooled CRISPR screen (2026) systematically targeting 23 NDD risk genes in human iPSC-derived neural progenitor cells, glutamatergic neurons, and GABAergic neurons provided the strongest experimental evidence for this principle (Fernandez Garcia et al., 2026). The study revealed that convergence points are greatest in mature glutamatergic neurons, affecting synaptic biology, epigenetic regulation, and—unexpectedly—mitochondrial biology, a hallmark of neurodegenerative processes. Critically, NDD risk genes were found to converge on a finite set of biological pathways, despite the continued expansion of identified risk loci, suggesting that diverse genetic etiologies funnel into shared downstream molecular cascades (Paulsen et al., 2022).

A cortex-wide transcriptomic study in Nature (2022) further demonstrated this overlap by profiling 725 samples spanning 11 brain regions from 49 individuals with idiopathic ASD and 54 controls, identifying 4,223 differentially expressed genes and 9,474 differentially expressed transcripts (Gandal et al., 2022). The transcriptomic dysregulation exhibited an anterior-to-posterior gradient—most severe in frontal and temporal cortices—mirroring the hierarchical cortical transcriptomic changes observed in AD. Convergent analysis of ASD cortical organoids additionally identified a master regulatory network module whose dysregulation affects neurogenesis, neurite growth, and synaptic biology through epigenetic mechanisms shared with neurodegenerative conditions (Gordon et al., 2026), while a 2025 integrative multiomics study of iPSC-derived neurons from familial AD (FAD) patients identified shared downstream molecular cascades across distinct PSEN1, PSEN2, and APP mutations (Valdes et al., 2025).

This review aims to do more than list shared features between ASD and AD. It emphasizes that NDDs and NDEs share overlapping molecular substrates in some, but not all, temporal disruptions of common biological networks, and that this overlap does not by itself establish a causal progression from developmental disruption to later neurodegeneration. Recognizing this continuum provides a useful framework for identifying patients with genetic NDDs who may be at higher risk for early neurodegeneration. It also supports the sharing of biomarkers and treatment strategies across disease categories and guides the development of interventions targeting common nodes with transdiagnostic therapeutic potential. In addition, a complementary perspective developed in this review—the heterochrony hypothesis of ASD—proposes that the phenotypic landscape of ASD can be understood as a selective delay or non-completion of normal neurodevelopmental trajectories, providing a mechanistic framework that directly connects the biology of early development to the later neurodegeneration risk described throughout this review.

## 2 Epidemiological and clinical evidence of overlap from human studies

### 2.1 Elevated dementia risk in ASD populations

Population-based studies suggest an increased burden of early-onset dementia among adults with ASD. Vivanti et al. (2021) reported a higher prevalence and incidence of early-onset dementia in adults with ASD younger than 65 years compared with the general population. Importantly, the study did not distinguish Alzheimer’s disease from other forms of dementia and did not specifically identify participants as having idiopathic versus syndromic ASD (Vivanti et al., 2021; Phillips et al., 2025). These findings should therefore be interpreted as evidence of an association between ASD and early-onset dementia broadly, rather than as evidence of a specific ASD–AD relationship. APOE-related effects in older autistic adults have been examined separately. The APOE ε4 allele has a deleterious effect on verbal learning and short-term memory in middle-aged and older (MA+) individuals, as shown by a study of MA+ autistic adults ages 40–71 years. By 2030, there will be about 700,000 older autistic adults in the United States with a recognized diagnosis. In exploratory analyses stratified by sex and diagnostic group, autistic males carrying APOE ε4 showed poorer verbal learning (*p* = 0.02) than autistic males who did not carry APOE ε4. The study suggests that MA + autistic males may be particularly susceptible to the effects of APOE ε4 on verbal learning, highlighting a compounded biological vulnerability to cognitive deterioration. However, future research with larger samples is needed to determine whether autistic females are similarly vulnerable (Harker et al., 2023).

A 2025 family study based on linked Swedish registers provides some of the first evidence supporting familial co-aggregation of autism and various forms of dementia, as well as a possible genetic relationship. Relatives of autistic people were more likely to develop dementia. The risk was highest among parents (hazard ratio [HR] = 1.36, 95% confidence interval [CI] = 1.25–1.49), and lower among grandparents (HR = 1.08, 95% CI = 1.06–1.10) and aunts/uncles (HR = 1.15, 95% CI = 0.96–1.38). Autism was more strongly associated with dementia in mothers (HR = 1.51, 95% CI = 1.29–1.77) than in fathers (HR = 1.30, 95% CI = 1.16–1.45) (Chang et al., 2025). These findings support familial co-aggregation between autism and dementia and are consistent with the contribution of familial factors to their association. However, this study design cannot disentangle inherited genetic effects from shared environmental or other familial influences and therefore does not establish a genetic causal relationship between the two conditions. Nevertheless, the findings provide population-level evidence supporting further investigation of shared familial determinants of neurodevelopmental and neurodegenerative vulnerability.

The association itself was not mechanistically tested in the familial data. A separate line of evidence concerns glymphatic function, indexed by DTI-ALPS (diffusion tensor imaging along the perivascular space), a neuroimaging measure of water diffusion along perivascular spaces and, by inference, waste-clearance capacity. DTI-ALPS-L, DTI-ALPS-R, and mean DTI-ALPS were all significantly lower in children with ASD than in typically developing controls. A similar reduction has been reported in AD dementia, in which impaired glymphatic clearance has been linked to amyloid-β accumulation and tau propagation (Wang et al., 2025a; Zhang Y. et al., 2025); however, this parallel is observational and does not establish a shared mechanism with the familial association. Independently, a randomized crossover trial in 39 participants found that glymphatic clearance during normal sleep increased morning plasma levels of AD biomarkers relative to sleep deprivation, consistent with sleep-dependent lowering of brain parenchymal resistance and overnight transfer of these biomarkers into plasma (Hu et al., 2023; Dagum et al., 2026).

### 2.2 Neurological regression in monogenic NDDs

As improved medical care has extended the lifespan of individuals with genetic neurodevelopmental disorders into middle and late adulthood, a new and clinically important phenomenon has emerged: progressive cognitive and neurological decline in aging NDD patients. A systematic review in Neurology (2024) examining dementia across 14 rare genetic neurodevelopmental disorders (RGNDs) identified 49 clinically confirmed cases of dementia, with an average onset age of 44.4 years. This onset is substantially earlier than the typical sporadic AD onset of 65–70 years, suggesting that existing neurodevelopmental molecular disruptions lower the threshold for neurodegeneration and accelerate its clinical expression.

The most frequently identified comorbid contributors to cognitive decline were epilepsy—which accelerates neuronal damage through excitotoxicity—followed by psychotic disorders and movement disorders. A critical finding was that diagnostic workup, including biomarker assessment, was not disclosed for 51% of reported individuals, highlighting a major clinical gap in the recognition and systematic evaluation of neurodegeneration in NDD populations. This gap not only delays diagnosis and intervention but also severely limits the epidemiological characterization of the NDD-NDE interface, underscoring the urgent need for longitudinal biomarker studies in aging NDD cohorts (Kwetsie et al., 2024).

### 2.3 Fragile X-associated tremor/ataxia syndrome (FXTAS) as a prototypical bridge causing neurodegeneration

Fragile X-associated tremor/ataxia syndrome (FXTAS) provides perhaps the most striking clinical evidence that a single gene, FMR1, can cause both neurodevelopmental and neurodegenerative disease, depending on the nature and severity of the mutation (Cabal-Herrera et al., 2020). While full CGG expansions (>200 repeats) cause FXS through transcriptional silencing and loss of FMRP, permutations (55–200 repeats) result in paradoxically different pathology: FMR1 mRNA is overproduced 3 to 8 fold, FMRP levels are only moderately reduced, and the excess mRNA forms intranuclear inclusions through an RNA toxic gain of function mechanism, driving progressive neurodegeneration in carriers older than 50 years (Figure 1) (Hagerman and Hagerman, 2013).

**Figure 1:**
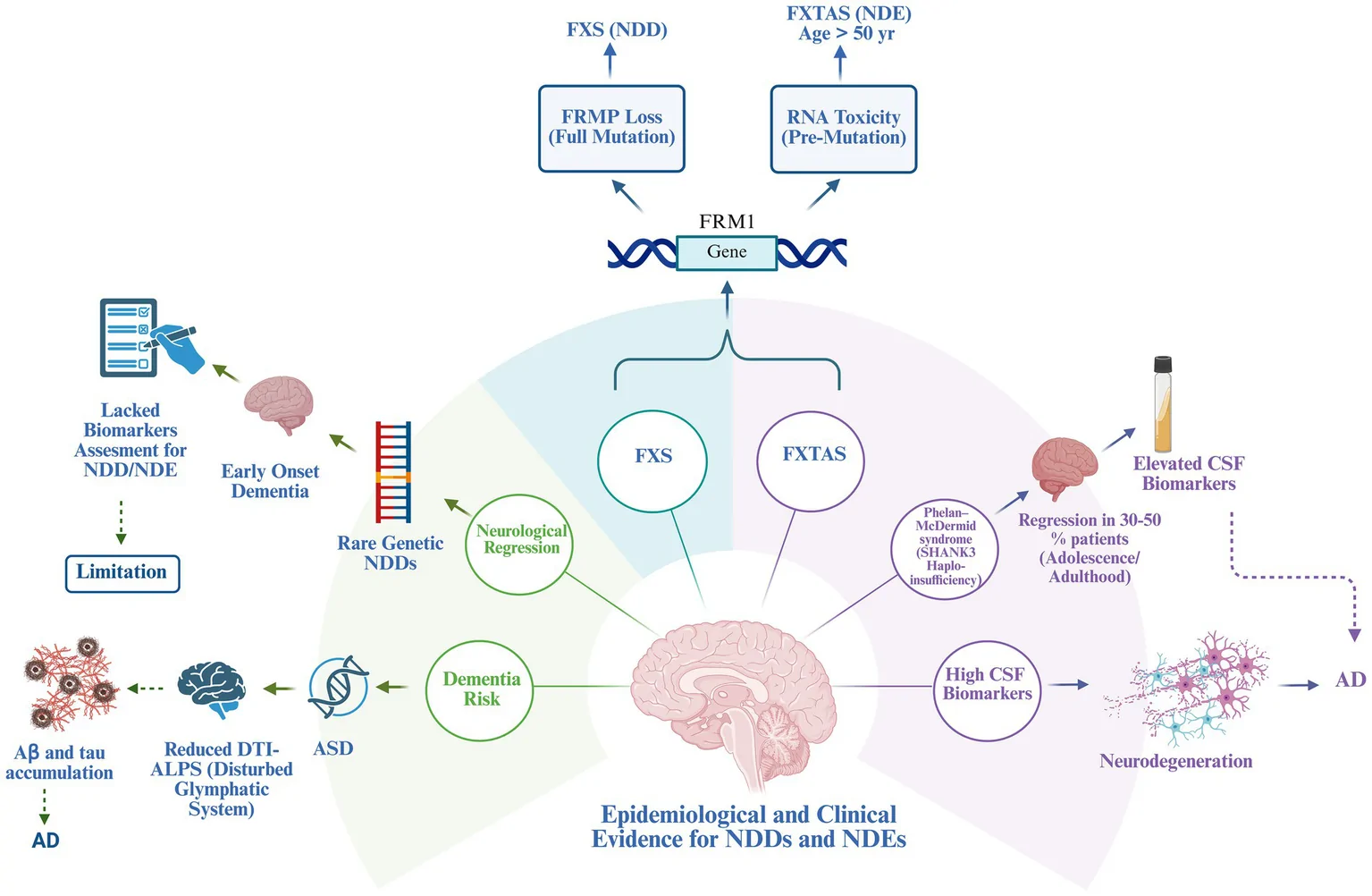
Epidemiological and clinical evidence from human studies linking ASD and AD risk, solid arrows denote associations directly reported in the cited studies; dashed grey arrows denote proposed links that have not been demonstrated longitudinally.

FXTAS is clinically characterized by progressive intention tremor, cerebellar gait ataxia, cognitive decline progressing to dementia, parkinsonism, peripheral neuropathy, and autonomic dysfunction. MRI reveals characteristic T2 hyperintensities in the middle cerebellar peduncles (“MCP sign”), progressive brain atrophy, and white matter lesions (Sellier et al., 2014). Approximately 40 of male and 16% of female premutation carriers develop FXTAS after age 50, with penetrance increasing with age and CGG repeat length (Cabal-Herrera et al., 2020). FXTAS thus serves as a unique human model system for studying the NDD-NDE transition, with measurable prodromal biomarkers and a defined molecular mechanism.

### 2.4 Phelan-McDermid syndrome regression and CSF biomarker signatures

Phelan-McDermid syndrome (PMS), caused by SHANK3 haploinsufficiency, provides another compelling clinical example of the NDD-NDE interface. Although PMS is classified as a neurodevelopmental disorder with onset in infancy, an estimated 30–50% of patients experience neurological regression—loss of previously acquired language, motor abilities, and adaptive behaviors—typically beginning in adolescence or early adulthood (Figure 1) (Dille et al., 2023).

A pivotal multimodal study (2023) examined whether this regression reflects static developmental stagnation or active neurodegeneration by combining cerebrospinal fluid (CSF) biomarker analysis with computational MRI in adult PMS patients (Jesse et al., 2023). CSF measurements of neurofilament light chain (NfL), glial fibrillary acidic protein (GFAP), and total tau—validated biomarkers of neurodegeneration, astrocyte activation, and neuronal injury—showed that a subset of adults with PMS and regression had elevated NfL and tau levels, consistent with ongoing neuronal injury and axonal degeneration. Computational MRI demonstrated progressive brain volume loss in cortical and subcortical regions, a pattern more consistent with active neurodegeneration than with a static developmental anomaly (Jesse et al., 2023).

Recent experimental studies have also identified molecular alterations associated with SHANK3 deficiency that overlap with pathways implicated in neurodegeneration; these mechanisms are discussed in detail in Section 3.1 (Varella-Branco et al., 2026).

## 3 Molecular and cellular convergences

### 3.1 Synaptic scaffolding, proteostasis and proteomic overlap

Synaptic dysfunction is the most consistent and well-documented feature shared by NDDs and NDEs, although the timing, direction, and molecular factors involved vary widely across conditions and genetic subtypes. A comprehensive proteomic comparison of five ASD mouse models (*Fmr1-KO*, *Cntnap2-KO*, *Pten+/−*, Anks1b+/−, and *BTBR+*) using quantitative mass spectrometry of hippocampal synaptic fractions revealed hundreds of proteins with shared alterations despite genetic differences. Key enriched pathways include oxidative phosphorylation, suggesting mitochondrial dysfunction at synapses, and Rho GTPase signaling, which regulates actin dynamics, spine morphology, and synaptic plasticity (Carbonell et al., 2023).

SHANK3 functions as a master scaffolding protein in the postsynaptic density (PSD) at glutamatergic synapses, organizing the PSD through a modular domain structure. A 2025 Cell study showed that SHANK3 oligomerization through its SAM domain governs the material properties of the PSD condensate and directly regulates synaptic plasticity, establishing that the structural organization of the PSD fundamentally depends on SHANK3 phase separation (Jia et al., 2025). This function is disrupted in PMS due to haploinsufficiency, and it is also progressively compromised in AD brains, where SHANK3 expression decreases as disease progresses (Varella-Branco et al., 2026).

Synaptic dysfunction also intersects with mGluR5- and complement-dependent mechanisms, providing an additional connection between developmental and degenerative synaptic pathology; the APP–FMRP–mGluR5 axis and its therapeutic implications are discussed in Section 7.2.

Collectively, SHANK3-dependent PSD organization serves as a key molecular convergence point. In ASD/PMS, haploinsufficiency disrupts PSD condensate stoichiometry from early development onward, leading to structurally modified yet stable synapses. Conversely, in AD, age-related decreases in SHANK3 expression weaken an already established PSD, resulting in synapse loss. The common downstream effect—impaired PSD condensate integrity—is less well understood than the phenomena described in later subsections. Current evidence does not clarify whether developmental changes in the PSD in ASD directly lead to faster PSD deterioration with age; this remains a critical area for future longitudinal studies.

### 3.2 mTOR signaling, autophagy and translational control

The mechanistic target of rapamycin (mTOR) signaling pathway is a central, convergent hub linking ASD and AD at multiple molecular levels (Lipton and Sahin, 2014; Xie et al., 2024). A bioinformatic analysis comparing ASD predisposition genes from the SFARI database with AD risk gene sets identified 148 genes shared between the two conditions, of which 75 (50.7%) are directly involved in mTOR signaling (Trifonova et al., 2025). This overlap should be interpreted as a shared regulatory architecture rather than direct evidence of a common causal pathway between the two conditions. Hub genes at this intersection—CTNNB1, CREBBP, TRAF2, HDAC1, and HDAC2—reflect the deep integration of mTOR signaling with synaptic plasticity, chromatin remodeling, and inflammatory signaling (Trifonova et al., 2025).

In ASD, mTOR hyperactivation during critical developmental windows produces aberrant neuronal connectivity, macrocephaly, and synaptic dysfunction—mechanisms well-characterized in tuberous sclerosis complex (TSC), PTEN hamartoma tumor syndrome, and neurofibromatosis type 1 (Drehmer et al., 2024). In AD, mTOR activation occurs early in the disease course and promotes Aβ and tau aggregation through two complementary mechanisms: increased protein synthesis, including APP and tau production, and reduced autophagy, which impairs the clearance of misfolded proteins (Xie et al., 2024). Strikingly, conditional knockout of Tsc1 specifically in microglia revealed a paradoxical protective effect—enhanced TREM2 expression and increased Aβ phagocytosis—that was completely abolished by rapamycin, demonstrating that mTOR inhibition has opposing effects in different cell types within the same brain (Shi et al., 2022). This cellular specificity underscores a major challenge for therapeutic targeting and illustrates the complexity of manipulating convergent pathways.

In this subsection, mTOR hyperactivation is presented as the convergent molecular node. In ASD, it drives excessive synaptic connectivity and suppresses autophagy during development; in AD, the same hyperactivation later impairs proteostatic clearance of Aβ and tau. The proposed link to later vulnerability is that the same molecular machinery—mTOR signaling and autophagy flux—that produces developmental synaptic excess in ASD may leave the aging brain less equipped to meet the proteostatic demands associated with AD, a hypothesis elaborated further in Section 7.6, although this sequential causal link has yet to be directly tested.

### 3.3 Calcium homeostasis and Wnt/β-catenin signaling

Calcium (Ca^2+^) is a universal second messenger that governs virtually every aspect of neuronal function, including neurotransmitter release, synaptic plasticity, gene expression, mitochondrial energetics, and cell survival (Brini et al., 2014). A review of the literature identified calcium signaling and molecular adhesion processes as core genetic risk pathways, with key regulators including SERCA2 (ATP2A2), ryanodine receptors (RyR1-3), inositol triphosphate receptors (ITPR1-3), and parvalbumin (PVALB) (Klocke et al., 2023). Parvalbumin interneuron dysfunction, driven in part by calcium signaling abnormalities, directly contributes to the excitatory/inhibitory (E/I) imbalance that is a hallmark of ASD (Filice et al., 2020).

PSEN1 and PSEN2—the genes responsible for most cases of familial AD—have well-characterized roles in ER Ca^2+^ homeostasis that are independent of their γ-secretase activity. FAD-PSEN2 mutants (e.g., T122R) reduce ER Ca^2+^ content by approximately 20%, as measured using the ER-targeted FRET-based Ca^2+^ sensor D4ER, and this ER Ca^2+^ depletion impairs autophagy at a specific step: it reduces RAB7 recruitment to autophagosomes, blocking autophagosome-lysosome fusion (Fedeli et al., 2019). The updated “calcium signaling hypothesis” of AD (2025) positions Ca^2+^ dysregulation as a central integrative mechanism that intersects with the amyloid cascade, tau hyperphosphorylation, neuroinflammation, and autophagy (Wang et al., 2025b), thereby linking the molecular pathophysiology of FAD with the calcium channel and receptor vulnerabilities documented in ASD.

The Wnt/β-catenin signaling pathway plays essential roles in cell specification, proliferation, polarity, neurogenesis, and synaptic plasticity throughout brain development and maintenance. CTNNB1, which encodes β-catenin, ranks among the top risk genes for NDDs and is among the most recurrently mutated genes in intellectual disability and ASD databases (Zhuang et al., 2023). A 2024 study in Nature demonstrated that the histone demethylase KDM5C regulates neurodevelopmental timing through Wnt signaling; KDM5C loss disrupts the timing of Wnt-dependent developmental processes and causes cognitive dysfunction, directly linking chromatin remodeling to temporal control of the Wnt pathway (Karwacki-Neisius et al., 2024).

In AD, Wnt/β-catenin signaling is progressively impaired by hyperactivation of GSK-3β (glycogen synthase kinase 3β), a negative regulator of β-catenin and one of the principal kinases responsible for tau hyperphosphorylation. When GSK-3β is hyperactivated, as in AD, it simultaneously disrupts Wnt signaling (through β-catenin degradation) and promotes tau pathology (through tau hyperphosphorylation), producing a dual pathological output from a single signaling node (Sai Varshini et al., 2024; Sharma et al., 2024). This makes GSK-3β an attractive transdiagnostic therapeutic target with the potential to concurrently address ASD-relevant neurodevelopmental timing, AD-relevant tau pathology, and synaptic plasticity deficits shared by both conditions.

Calcium signaling and Wnt/β-catenin regulation are two interconnected pathways that produce distinct cellular effects. In ASD, disruptions in Ca^2+^ handling and alterations in CTNNB1/KDM5C primarily affect E/I balance and developmental timing. In contrast, in AD, ER Ca^2+^ depletion impairs autophagy, and overactivation of GSK-3β leads to tau hyperphosphorylation. As noted earlier, the cellular outcomes in these conditions diverge substantially, and any proposed role in subsequent vulnerability should be considered preliminary rather than conclusive.

## 4 Neuroinflammation and immune pathways

### 4.1 Innate immunity: microglia, TREM2, and NLRP3 inflammasome

Neuroinflammation, once considered a secondary bystander in neurological disease, is now recognized as a central driver of pathology in both NDD and NDE (Mottahedin et al., 2017). Postmortem studies document chronic microglial activation in ASD brains, while CSF and plasma analyses reveal elevated proinflammatory cytokines—IL-6, TNF-α, IL-1β, and MCP-1—in some studies, correlating with behavioral severity (Figure 2) (Liao et al., 2020). This chronic, low-grade neuroinflammation beginning in early development disrupts synaptic pruning, alters neurotransmitter homeostasis, and creates an oxidative environment that exacerbates epigenetic and metabolic dysfunction.

**Figure 2:**
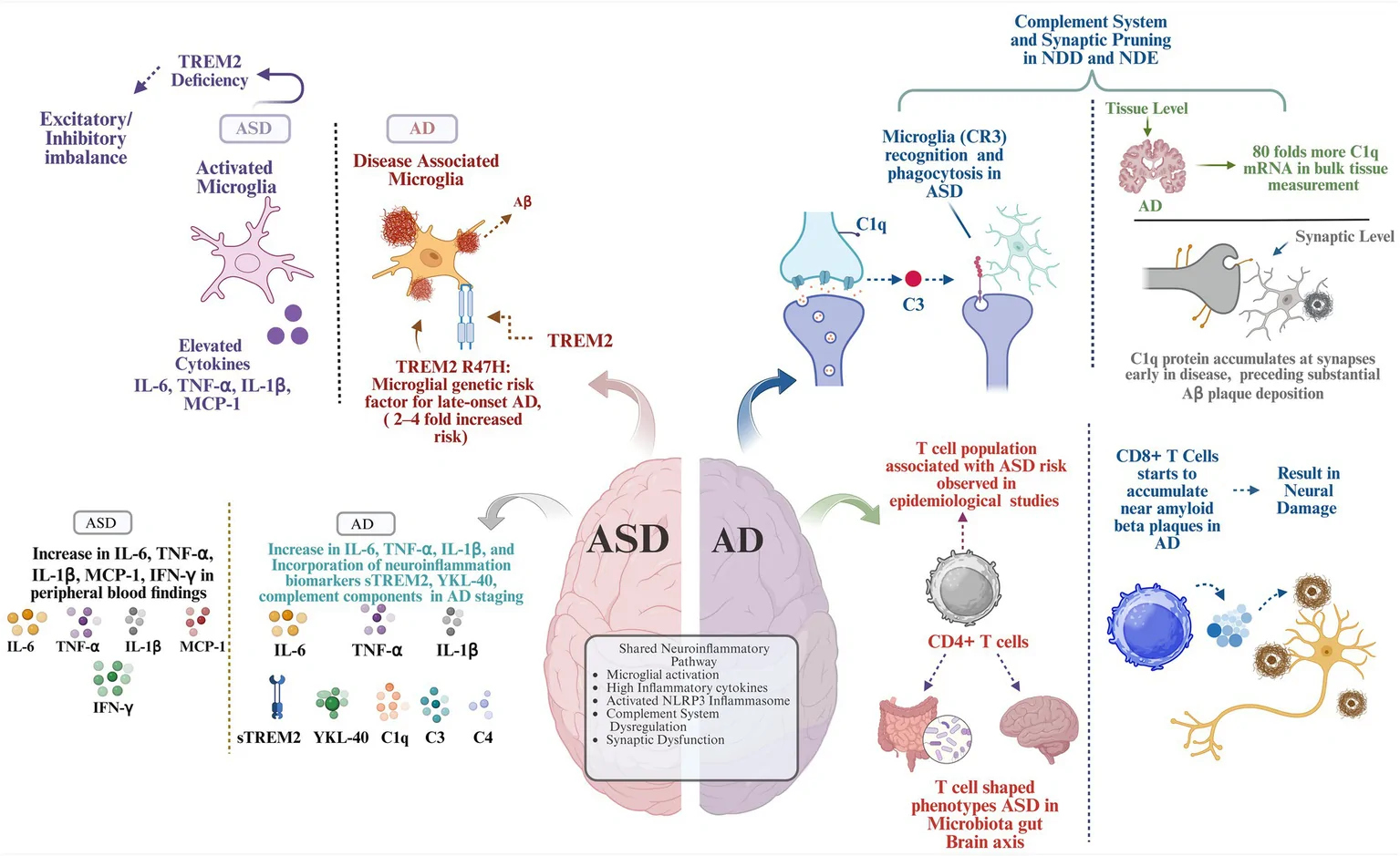
Shared and divergent immune pathways and neuro-inflammation.

In AD, single-cell RNA sequencing studies have characterized “disease-associated microglia” (DAM)—a stereotyped transcriptomic transition of microglia near Aβ plaques from homeostatic to activated states that is critically dependent on TREM2 (triggering receptor expressed on myeloid cells 2) (Keren-Shaul et al., 2017). The R47H variant of TREM2, the strongest known microglial genetic risk factor for late-onset AD, confers a 2–4-fold increased risk and impairs ligand binding and downstream signaling (Gratuze et al., 2020). Notably, evidence from 2025 showed that TREM2 deficiency produces an excitatory/inhibitory (E/I) imbalance—a hallmark neurophysiological feature of ASD—thereby establishing TREM2 as a convergent microglial regulator in both conditions (Wang Y. et al., 2025) (Table 1).

**Table 1:** Immune pathway dysregulation across ASD and AD.

Pathway	ASD	AD	Convergence	References
Microglia	Chronic activation; TLR2/4, NLRP3, NF-κB	DAM phenotype; TREM2-tau loop; NLRP3-caspase-1	TREM2 deficiency causes E/I imbalance	Mottahedin et al. (2017); Keren-Shaul et al. (2017); Wang Y. et al. (2025)
Complement	Bidirectional (excess or insufficient pruning)	C1q mRNA: up to ~80-fold increase; early synaptic C1q protein accumulation; C3/CR3 spatial heterogeneity	mGluR5 SAM prevents C1q synaptic tagging	Yasojima et al. (1999); Hong et al. (2016); Stevens et al. (2007); Ye et al. (2026)
T cells	CD8bright↑; CD28^−^CD8dim↑; CD4^+^ gut-brain	CD8^+^ infiltrate brain; perforin/granzyme; Treg protective	Shared genetic regulation (PTEN, MTOR, TSC1)	Yu et al. (2024); Park et al. (2025); Terrabuio et al. (2025)
Cytokines	IL-6, TNF-α, IL-1β, MCP-1, IFN-γ	IL-6, TNF-α, IL-1β; sTREM2, YKL-40	Overlapping inflammatory profiles; neuro-immune activation in both conditions	Liao et al. (2020); Mottahedin et al. (2017); Jin et al. (2025)
AhR	Cinnabarinic acid → chronic AHR activation	KP metabolite-mediated AHR regulation	KP-AHR axis convergence	Launay et al. (2023)

DAM, disease-associated microglia; E/I, excitatory/inhibitory; AHR, aryl hydrocarbon receptor; STING, stimulator of interferon genes.

It is important to note that neuroimmune alterations do not necessarily play the same causal role in the two conditions. In ASD, current evidence does not clearly establish whether inflammatory and microglial changes represent primary developmental drivers, secondary adaptations, or biologically distinct subgroups. For instance, maternal immune activation has been associated with increased ASD risk; however, genetic factors complicate the interpretation of this association (Han et al., 2021; Gardner et al., 2025). In AD, by contrast, immune mechanisms have stronger evidence as contributors to disease progression, although they remain stage- and context-dependent (Heneka et al., 2025; Weiner, 2025). This distinction should be kept in mind when interpreting neuroimmune convergence between ASD and AD and is reiterated in the complement and adaptive immunity discussions below.

### 4.2 Complement cascade and developmental vs. pathological synaptic pruning

The classical complement cascade—C1q → C4 → C2 → C3 → membrane attack complex—plays dual roles in the brain: it supports essential developmental synaptic pruning and, when aberrantly reactivated, drives pathological synapse destruction (Stevens et al., 2007; Ye et al., 2026). During early postnatal development, C1q is deposited on synapses destined for elimination, and microglia expressing the C3 receptor (CR3) engulf C3-opsonized synapses through phagocytosis (Figure 2) (Stevens et al., 2007). Dysregulation of this system—whether through excessive pruning, which contributes to reduced synaptic density in ASD, or insufficient pruning, which leads to excess immature synapses—represents a key locus of complement-mediated pathology in neurodevelopmental conditions.

In AD, the developmental complement system is aberrantly reactivated. C1q mRNA has been reported to increase by as much as 80-fold in vulnerable brain regions, including the hippocampus (Yasojima et al., 1999), while C1q protein accumulates at synapses early in disease progression, preceding substantial amyloid plaque deposition and supporting an early role for complement-mediated synapse elimination (Hong et al., 2016). A 2026 study showed that microglia-complement interactions differ across brain regions, with the effectiveness of complement-mediated synapse removal varying by region, introducing spatial heterogeneity into a process previously assumed to be uniform (Ye et al., 2026). The 2022 demonstration that the mGluR5 SAM BMS-984923 prevents C1q synaptic localization and fully restores synapse density in aged AD mice provides a mechanistic bridge linking mGluR5 overactivation in FXS to complement-mediated synapse loss in AD, and positions mGluR5 modulation as a transdiagnostic anti-complement strategy (Spurrier et al., 2022).

The key convergence point is the C1q–C3 complement pruning cascade. In ASD, abnormal developmental pruning—either excessive or insufficient—leads to lasting structural changes in synapses. In contrast, in AD, inappropriate reactivation of this same cascade causes synapse loss in mature circuits. This machinery, used for opposite purposes—shaping circuits versus destroying them—clearly illustrates how a shared molecular pathway can produce different cellular outcomes. Additionally, inadequate developmental pruning might make the aging brain less able to properly regulate complement-tagged synapse elimination, although this specific sequence has not yet been directly demonstrated.

### 4.3 Adaptive immunity: T cells and B cells and peripheral cytokine signatures

The role of adaptive immunity in brain disorders has traditionally been underappreciated; however, recent evidence has revealed substantial T cell involvement in both ASD and AD (Yu et al., 2024; Park et al., 2025). A 2024 Mendelian randomization study evaluating 731 immune cell phenotypes for causal relationships with ASD risk identified several T cell populations with significant associations, particularly CD8bright and CD28^−^CD8dim subsets (Yu et al., 2024). A landmark 2025 Nature Communications study demonstrated that the gut microbiota and brain-resident CD4^+^ T cells jointly shape ASD phenotypes through the microbiota-gut-brain axis, thereby establishing bidirectional communication between peripheral immunity and central nervous system function as a driver of ASD-relevant behavioral traits (Park et al., 2025).

In AD, CD8^+^ T cells infiltrate the brain parenchyma, accumulate near amyloid plaques and neurofibrillary tangles, and directly mediate neuronal damage through perforin/granzyme pathways and Fas/FasL interactions (Terrabuio et al., 2025). The convergence of T cell dysregulation in ASD and AD, involving shared genetic regulation, suggests that adaptive immune modulation may represent a transdiagnostic therapeutic strategy, particularly given the roles of these pathways in immune cell trafficking and neuronal function.

Circulating cytokine profiles overlap substantially between ASD and AD, reflecting shared neuroinflammatory mechanisms despite the distinct temporal contexts of their activation (Liao et al., 2020; Terrabuio et al., 2025). In ASD, peripheral blood consistently shows elevated levels of IL-6, TNF-α, IL-1β, MCP-1, and IFN-γ, with cytokine levels correlating with behavioral severity across multiple cohorts (Liao et al., 2020). In AD, peripheral inflammation mirrors CNS neuroinflammation: elevated plasma IL-6, TNF-α, and IL-1β are well documented, and the proposed ATN-X framework incorporates neuroinflammatory biomarkers (sTREM2, YKL-40, complement components) into AD biological staging (Jin et al., 2025).

In ASD, this developmental pattern manifests as impaired synaptic energetics affecting key clinical domains; in AD, mitochondrial dysfunction precedes and amplifies amyloid and tau pathology (Uittenbogaard and Chiaramello, 2014). The similar oxidative biomarker patterns suggest that early deficits in metabolic and redox reserves may reduce resilience to the energetic and proteostatic stresses of aging, although this potential link to later vulnerability has not yet been tested.

## 5 Metabolic, mitochondrial and clearance pathways

### 5.1 Mitochondrial dysfunction, oxidative stress and energy metabolism

Mitochondrial dysfunction connects NDDs and NDEs at the most fundamental level of cellular energy metabolism and is among the most robustly identified convergent features across the NDD–NDE spectrum (Uittenbogaard and Chiaramello, 2014). A 2026 CRISPR NDD convergence study provided the strongest mechanistic evidence: mitochondrial function was among the most prominently affected convergent networks across 23 NDD risk genes in mature glutamatergic neurons, positioning mitochondrial vulnerability as a key driver of shared pathophysiology (Fernandez Garcia et al., 2026). The 2025 Molecular Psychiatry review by Khaliulin et al. (2025) presented the most comprehensive case for mitochondrial dysfunction in ASD, assembling evidence spanning electron transport chain (ETC) dysfunction across complexes I–V, aberrant mitochondrial Ca^2+^ handling, excessive ROS/RNS production, and dysregulated mitochondrial permeability transition pore (mPTP) opening.

In AD, mitochondrial dysfunction precedes amyloid deposition by years and represents one of the earliest detectable molecular events, making it a potentially valuable target for presymptomatic intervention (Uittenbogaard and Chiaramello, 2014). A 2025 review established that AD mitochondrial pathology encompasses imbalances in mitochondrial dynamics (fusion/fission), defective mitophagy mediated by PINK1/Parkin, impaired bioenergetics, and accumulation of mtDNA damage, with Aβ oligomers directly disrupting mitochondrial membrane integrity through cyclophilin D (CypD) interactions (D’Alessandro et al., 2025). The convergence of NDD and NDE mitochondrial pathology on ETC complex I–III dysfunction, Ca^2+^ mishandling, and mitophagy impairment suggests that mitochondria-targeted therapies—including CoQ10 analogs, PINK1/Parkin activators, and novel ETC modulators—may have transdiagnostic therapeutic value.

Oxidative stress, defined as an imbalance between the production of reactive oxygen and nitrogen species (ROS/RNS) and the capacity of antioxidant defenses, is both a consequence and an amplifier of mitochondrial dysfunction and is consistently documented across the NDD-NDE spectrum. A comprehensive 2025 review concluded that glutathione and its derivatives (GSH, GSSG, GSH/GSSG ratio) and isoprostanes (F₂-isoprostanes) are the most promising oxidative stress biomarkers for ASD diagnosis, Blood-based studies consistently report decreased antioxidant enzyme activities (SOD, GPx, catalase), elevated lipid peroxidation markers (MDA, 4-HNE, F₂-isoprostanes), and elevated protein oxidation markers (protein carbonyls, 3-nitrotyrosine). The regions with the highest oxidative burden in ASD—the prefrontal cortex, temporal cortex, and cerebellum—correspond to areas involved in core clinical features of social communication, language, and sensorimotor function (Kuźniar-Pałka, 2025).

In AD, oxidative damage is a defining neuropathological feature, with Aβ-driven membrane oxidative stress, 8-OHdG accumulation, and protein carbonyl accumulation documented in affected brain regions. The overlapping oxidative stress biomarker profile between ASD and AD suggests shared redox dysregulation mechanisms and positions antioxidant strategies, particularly those targeting the GSH system and isoprostane pathway, as cross-condition interventions. Nonenzymatic FAD oxidation by reactive oxygen species also directly impairs histone demethylase activity, providing a mechanistic link between oxidative stress and the epigenetic dysregulation (Niu et al., 2015).

### 5.2 Tryptophan–kynurenine, sphingolipid and purine metabolism

Tryptophan (TRP), an essential amino acid, is the precursor for two major metabolic branches: serotonin (5-HT) synthesis (~5% of TRP metabolism) and the kynurenine pathway (KP, ~95% of TRP metabolism), which generates a series of neuroactive metabolites that modulate glutamatergic transmission, immune responses, and redox balance (Badawy, 2017; Holeček, 2026). In a landmark cohort study of 271 individuals with ASD, Launay et al. (2023) reported that indoleamine 2,3-dioxygenase (IDO—the rate-limiting enzyme that diverts TRP toward the KP—was activated in 58.7% (159/271) of participants. This IDO activation directly suppressed serotonin synthesis: individuals with IDO activation tended toward normoserotonemia, whereas those without IDO activation showed the expected hyperserotonemia. The authors estimated that hyperserotonemia could be present in approximately 94% of individuals with ASD in the absence of IDO activation (Launay et al., 2023).

KP perturbation in AD has been extensively characterized using CSF and serum metabolomics. In a clinical study comparing 33 AD patients, 33 PD patients, and 39 age-matched controls, kynurenic acid—the neuroprotective branch metabolite—was markedly reduced in the CSF of both AD and PD patients (*p* < 0.002 for both) (Sorgdrager et al., 2019). Plasma TRP levels were negatively correlated with CSF total tau (*r* = −0.19), and kynurenine (KYN) with CSF phospho-tau (r = −0.18), establishing quantitative links between KP metabolite levels and tau pathology severity (Bakker et al., 2023). The shared but directionally distinct KP dysregulation across ASD and AD—IDO hyperactivation in ASD versus kynurenic acid depletion in AD—suggests that targeting KP metabolic branch (IDO modulation, kynurenine aminotransferase enhancement, QPRT activation) represents a precision medicine strategy with transdiagnostic potential.

Sphingolipids account for approximately 20–25% of total brain lipid content and are essential structural and signaling components of neuronal membranes (Hussain et al., 2019). A comprehensive lipidomic study of AD brains revealed significantly elevated sphingosine (Sph), total ceramide (Cer), total ceramide 1-phosphate (Cer1P), and deoxy-ceramide compared to age-matched controls. Mechanistically, Aβ peptides induce membrane-associated oxidative stress that perturbs ceramide and cholesterol metabolism: Aβ42 activates neutral sphingomyelinase (nSMase), which hydrolyzes sphingomyelin to ceramide in the outer leaflet of the plasma membrane, thereby triggering pro-apoptotic ceramide signaling (Uranbileg et al., 2024).

A 2025 comprehensive review established that precise regulation of sphingolipid metabolism is essential during cortical development and neurogenesis (Chinnappa et al., 2025). ASD patients with autistic regression showed specific dysregulation of sphingolipid metabolism, distinguishing them from non-regressive ASD (Yan et al., 2025). A 2023 metabolic modeling study identified sphingolipid metabolism as one of three core dysregulated metabolic subsystems in ASD (Table 2), alongside neuroinflammation and energy metabolism (Esvap and Ulgen, 2023). The convergent ceramide accumulation pathology in AD and ASD—though arising through distinct mechanisms—suggests that sphingomyelin synthesis inhibitors and ceramidase activators warrant investigation across the NDD-NDE continuum.

**Table 2:** Metabolic pathway dysregulation across ASD and AD.

Pathway	ASD	AD	Convergence evidence	References
Tryptophan-KP	IDO activation (58.7%); kynurenate↓; NAD↓; miR-153-3p	KA↓ in CSF; QA↑; plasma KYN inversely correlates with p-tau	KP dysregulation; fecal-brain TRP metabolite-fMRI link	Launay et al. (2023); Sorgdrager et al. (2019); Bakker et al. (2023)
Gut microbiome	PPA↑; butyrate↓; AKT1/IL6 hub; PI3K-Akt/IL-17	SCFAs promote Aβ in GF mice; Firmicutes/Bacteroidetes↓	Microbiome-brain axis; CD4^+^ T cells mediate gut-behavior	Zhang F. et al. (2025); Colombo et al. (2021); Park et al. (2025); Kowalski and Mulak (2019)
Sphingolipids	Brain sphingolipid dysregulation; autistic regression subtype	Ceramide↑, Sph↑, Cer1P↑; nSMase activation by Aβ	Sphingolipid metabolism as core dysregulated subsystem	Esvap and Ulgen (2023); Yan et al. (2025); Uranbileg et al. (2024); Chinnappa et al. (2025)
Purines	Adenosine/inosine/hypoxanthine altered; P2RX7, ADA dysregulated	Region-specific (frontal↓); plaque-independent; uric acid U-shaped	Purinergic signaling → NLRP3 inflammasome activation	Dai et al. (2023); Erb et al. (2019); Cieślak and Wojtczak (2018)
Mitochondria	ETC I-V↓; Ca^2+^↑; ROS↑; mPTP↑	ETC↓; mitophagy↓ (PINK1/Parkin); Aβ-CypD interaction	CRISPR screen convergence on mitochondrial networks	Fernandez Garcia et al. (2026); Khaliulin et al. (2025); D’Alessandro et al. (2025); Uittenbogaard and Chiaramello (2014)

KP, kynurenine pathway; IDO, indoleamine 2,3-dioxygenase; KA, kynurenic acid; QA, quinolinic acid; PPA, propionic acid; SCFAs, short-chain fatty acids; GF, germ-free; Cer, ceramide; Sph, sphingosine; nSMase, neutral sphingomyelinase; ETC, electron transport chain; mPTP, mitochondrial permeability transition pore; CypD, cyclophilin D.

Purines—adenosine, guanosine, inosine, hypoxanthine, xanthine, and uric acid—play dual roles in the brain as nucleic acid building blocks and energy carriers (ATP, GTP), as well as extracellular signaling molecules that act through purinergic receptors (Dai et al., 2023). Multi-omics study integrating metabolomics and transcriptomics confirmed dysfunction of the purine signaling pathway in ASD, with altered levels of adenosine, inosine, and hypoxanthine, accompanied by dysregulated expression of purinergic receptors—particularly P2RX7 (an ATP-gated cation channel that activates the NLRP3 inflammasome) and P2RY1 (Dai et al., 2023). The “extracellular ATP three-hit model” of ASD, in which excess eATP drives sustained P2RX7–NLRP3 activation, neuroinflammation, and synaptic dysfunction, provides a mechanistic link between purine metabolism and innate immune activation (Dai et al., 2023). Multiple studies confirm that purinergic signaling is disrupted in Alzheimer’s disease, spanning receptor dysfunction, metabolic alterations, and neuroinflammatory cascades (Cieślak and Wojtczak, 2018; Erb et al., 2019). The overlapping disruption of purinergic signaling in ASD and AD, converging on P2RX7-mediated NLRP3 inflammasome activation, positions purinergic signaling as a convergent therapeutic target amenable to pharmacologic modulation.

### 5.3 Gut microbiome-brain axis and short-chain fatty acids

The microbiota-gut-brain axis is increasingly recognized as a central regulatory system in both NDDs and NDEs, mediated through metabolite production, immune modulation, and vagal nerve signaling. A 2025 network pharmacology study that integrated ASD-related gene networks with gut microbiota metabolite target networks identified 51 core targets at the intersection, with AKT1 and IL6 as pivotal hub genes, and eight key metabolites—including short-chain fatty acids (SCFAs: acetate, butyrate, propionate) and indole derivatives—linked to their regulation. Propionic acid (PPA) has been consistently reported at elevated levels in ASD and can reproduce autism-like behavioral and neuroinflammatory phenotypes when administered to rodents, establishing a direct metabolite-behavior link (Zhang F. et al., 2025).

A key eLife study (2021) reported an unexpected finding: microbiota-derived SCFAs promote cerebral Aβ accumulation. Germ-free APP/PS1 mice showed a substantial decrease in Aβ plaques compared with conventionally housed mice, and administering SCFAs to germ-free mice reinstated amyloid buildup, indicating that gut metabolites can directly affect brain amyloid formation (Colombo et al., 2021). A 2019 review on microbiome-related AD treatments noted that transplanting fecal microbiota from healthy donors into AD mice lowers amyloid levels, and that probiotics—especially Lactobacillus and Bifidobacterium strains—as well as prebiotics, show promising results in early experiments and initial clinical trials (Kowalski and Mulak, 2019). This shared connection between gut dysbiosis and SCFA activity in both ASD and AD suggests a promising transdiagnostic treatment approach.

### 5.4 Glymphatic and waste-clearance mechanisms

Evidence of altered glymphatic-related function in ASD and AD has been investigated using the DTI-ALPS (diffusion tensor imaging along the perivascular space) index, an indirect neuroimaging measure associated with fluid movement along perivascular spaces. DTI-ALPS-L, DTI-ALPS-R, and mean DTI-ALPS were significantly reduced in children with ASD compared with typically developing controls, while reduced DTI-ALPS has also been reported in patients with AD dementia (Wang et al., 2025a; Zhang Y. et al., 2025). In addition, a randomized crossover study involving 39 participants found that normal sleep was associated with higher morning plasma concentrations of AD biomarkers than sleep deprivation, consistent with sleep-dependent clearance of brain-derived biomarkers (Hu et al., 2023; Dagum et al., 2026).

Genetic evidence further suggests that interindividual variation in glymphatic-related function may have a heritable component. Variants at the 2p23.3 locus have been associated with measures of glymphatic activity, raising the possibility that common biological determinants of brain clearance may operate across different stages of life (Huang et al., 2025). Together with the DTI-ALPS findings, these observations suggest that altered waste-clearance mechanisms may represent a potential point of biological convergence between neurodevelopmental and neurodegenerative conditions. However, they do not establish that glymphatic dysfunction during neurodevelopment causes, or directly predisposes to, subsequent AD-like neurodegeneration.

Glymphatic dysfunction should therefore be considered a candidate shared vulnerability rather than an established longitudinal pathway linking ASD and AD. Whether early alterations in clearance-related processes influence later susceptibility to amyloid, tau, or other age-related neuropathological changes will require prospective longitudinal investigation (Vivanti et al., 2021; Zhang Y. et al., 2025). This possibility can also be considered within the heterochrony framework developed in Section 7.6, without implying that such a developmental-to-degenerative trajectory has yet been demonstrated.

## 6 Epigenetic mechanisms

### 6.1 DNA methylation: DNMT3A, MeCP2, and the Rett-AD connection

MeCP2 (methyl-CpG binding protein 2) recognizes both CpG and CpH methylation marks and is mutated in Rett syndrome (RTT), one of the most common causes of severe intellectual disability in females. RTT provides a remarkable clinical bridge between NDDs and NDEs: after apparently normal early development (6–18 months), patients undergo rapid regression with loss of acquired speech, purposeful hand skills, and social engagement—followed by a long stable period and, in many cases, progressive motor and cognitive decline. MeCP2’s multilayered functions—as a transcriptional repressor recruiting the NCoR/SMRT co-repressor complex containing HDAC3, as a transcriptional activator through CREB1 interaction, as a chromatin architectural protein, and as a regulator of alternative splicing—position it as a master coordinator of neuronal gene expression programs that are compromised in both NDDs and in aging (Liu et al., 2025).

DNMT3A mutations cause Tatton-Brown-Rahman syndrome, a neurodevelopmental disorder with intellectual disability, and the same enzyme is progressively dysregulated in AD, where somatic DNMT3A mutations and altered DNMT3A activity contribute to age-related epigenomic drift. The MeCP2-BDNF-SIRT1 axis—in which MeCP2 regulates BDNF expression, BDNF activates SIRT1, and SIRT1 in turn modulates MeCP2 activity—represents a convergent regulatory loop whose disruption impairs neuronal survival and plasticity in both Rett syndrome and AD-related neurodegeneration (Liu et al., 2025).

Convergent node: MeCP2-dependent transcriptional regulation. In Rett syndrome, loss of MeCP2 function disrupts gene expression beginning early in development. In Alzheimer’s disease, a similar epigenomic drift associated with DNMT3A emerges progressively with aging. The shared MeCP2–BDNF–SIRT1 loop suggests a potential convergence point, but it remains unproven whether early MeCP2 dysfunction increases susceptibility to the age-related epigenomic changes observed in AD. This hypothesis warrants further investigation.

### 6.2 Histone acetylation: HDACs, HATs, and H3K27ac remodeling

Class I HDACs (HDAC1, HDAC2, HDAC3, HDAC8) are essential regulators of neural development, and their dysfunction is documented across multiple NDDs and NDEs. In mature neurons, HDAC2 accumulates at the promoters of memory-related genes (BDNF, CREB, CaMKIIα) and suppresses their expression; genetic deletion or pharmacological inhibition of HDAC2 enhances memory formation (Guan et al., 2009). HDAC3 displays regional and circuit-specific activity: selective inhibition with RGFP966 accelerates extinction memory consolidation through distinct histone acetylation patterns across brain regions, demonstrating that HDAC selectivity—rather than broad deacetylase inhibition—is required for therapeutic precision (Malvaez et al., 2013).

AD brains show extensive reconfiguration of H3K27ac at enhancers and super-enhancers, reflecting the epigenomic response to neurodegeneration. The HATs p300 and CBP (CREBBP) drive compensatory gene expression programs in AD-affected neurons: they increase H3K27ac at stress-response and inflammatory gene enhancers while H3K27ac is lost at synaptic and neuronal identity gene enhancers, creating a progressive redistribution of the acetylome that silences neuronal identity while amplifying neuroinflammatory programs (Nativio et al., 2020).

Convergent node: Regulation of the neuronal acetylome by Class I HDACs/HATs. In neurodevelopmental disorders (NDDs), dysregulation of HDAC2/HDAC3 disrupts memory and extinction gene programs during development and adulthood. In Alzheimer’s disease (AD), p300/CBP-driven redistribution of H3K27ac silences genes associated with neuronal identity and enhances inflammatory processes. These outcomes are related but involve distinct mechanisms of chromatin acetylation dysregulation. The authors emphasize that this convergence reflects a shared vulnerability of the acetylome, rather than a direct causal link between developmental and degenerative acetylation changes.

### 6.3 Histone methylation: G9a/EHMT2, KDM5C, and KMT2D

G9a (EHMT2) catalyzes mono- and dimethylation of H3K9, generating H3K9me1 and H3K9me2, marks associated with transcriptional repression at euchromatic regions G9a upregulation and H3K9me2 accumulation have been documented in the brains of AD patients and correlate with disease severity (Bellver-Sanchis et al., 2024). A 2025 Nature Communications study reported a brain-penetrant G9a methylase inhibitor that significantly reduced both Aβ and tau proteopathology *in vivo* when administered systemically to AD mouse models (Xie et al., 2025). A comprehensive November 2025 review in Medicinal Research Reviews synthesized evidence for G9a’s role across ASD, PWS, schizophrenia, epilepsy, anxiety, depression, and AD, establishing G9a as the most extensively validated cross-cutting epigenetic target for neuropsychiatric disorders—an extraordinary convergent therapeutic target bridging the NDD-NDE spectrum (Hajar et al., 2025).

KDM5C (an H3K4me2/H3K4me3 demethylase mutated in X-linked intellectual disability and ASD) regulates transcriptional timing of Wnt pathway genes during cortical development (Karwacki-Neisius et al., 2024), while KMT2D (MLL4) catalyzes H3K4 mono- and dimethylation at enhancers. A 2025 study showed that KMT2D deficiency specifically disrupts hippocampal E/I balance by reducing excitatory synaptic transmission onto GABAergic interneurons—a mechanism directly relevant to ASD E/I imbalance and the circuit-level vulnerability underlying AD-associated memory impairment (Shangguan et al., 2025).

Convergent node: G9a/EHMT2-driven H3K9 methylation. In ASD, decreased Tier 3–4 methyltransferase activity (see Section 7.3) is linked to epigenetic immaturity at synaptic gene loci; in AD, G9a upregulation and H3K9me2 accumulation indicate an overcompensatory repressive state that correlates with disease severity. This bidirectional dysregulation—reduced activity in ASD and increased activity in AD—constitutes a plausible but largely correlative point of convergence. It also highlights G9a as a potential universal therapeutic target, without implying that ASD-related epigenetic immaturity directly causes the AD-related hypermethylated state.

### 6.4 Chromatin remodeling and non-coding RNAs

CHD8 (chromodomain helicase DNA-binding protein 8) is among the top ASD risk genes, encoding an ATP-dependent chromatin remodeler that influences nucleosome placement at gene promoters. CHD8^+/−^ mice exhibit macrocephaly, altered cortical lamination, social behavior deficits, and changes in peripheral immune responses, suggesting that CHD8 coordinates both neural and immune developmental programs—linking chromatin remodeling to neuroinflammatory vulnerability (Katayama et al., 2016; Shi et al., 2023). Functionally, many autism-associated chromatin remodeling mutations—including CHD8, ARID1B, and ADNP—converge on regulation of neuronal identity genes and synaptic scaffolding gene enhancers, creating a common epigenetic endpoint despite diverse genetic origins.

*miR-153-3p*, a microRNA that negatively regulates IDO transcription, shows a strong negative correlation with the KYN/TRP ratio in ASD—linking non-coding RNA regulation to dysfunction of the tryptophan metabolic pathway and illustrating how ncRNA dysregulation coordinates epigenetic and metabolic convergence (Launay et al., 2023) (Table 3).

**Table 3:** Dysregulated epigenetic modifications across neurodevelopmental and neurodegenerative disorders.

Modification	ASD/NDDs	AD	Convergence	References
DNA methylation	MeCP2 (Rett syndrome); DNMT3A mutations	DNMT3A↑; MeCP2 in aging	MeCP2-BDNF-SIRT1 axis	Liu et al. (2025)
H3K27ac (HATs)	CREBBP (Rubinstein-Taybi); CBP/p300	H3K27ac reconfiguration; CBP/p300 compensatory changes	CREBBP as an ASD-AD hub gene	Nativio et al. (2020)
H3K9me2/me3 (G9a)	G9a/SETDB1/ZNF274 → SNORD116 silencing (PWS)	G9a↑; H3K9me2↑; brain-penetrant G9a inhibitors reduce Aβ/tau	G9a as a cross-cutting epigenetic target	Bellver-Sanchis et al. (2024); Bellver-Sanchis et al. (2025); Xie et al. (2025)
H3K4me2/me3	KDM5C (X-linked ID); KMT2D (Kabuki syndrome)	KDM5C in aging; H3K4me3 at inflammatory genes	Balanced H3K4 methylation is essential for cognition	Karwacki-Neisius et al. (2024); Shangguan et al. (2025)
HDACs	HDAC1/2 (neural development); HDAC3 (memory extinction)	HDAC2 (memory suppressor); class I HDACs in neurodegeneration	HDACs as validated therapeutic targets	Guan et al. (2009); Malvaez et al. (2013)
Non-coding RNA	miR-153-3p	miR-153-3p → IDO regulation	miRNA dysregulation; ncRNA-epigenetic-metabolic crosstalk	Launay et al. (2023)

MeCP2, methyl-CpG binding protein 2; DNMT3A, DNA methyltransferase 3A; HATs, histone acetyltransferases; CBP, CREB-binding protein; PWS, Prader-Willi syndrome; ID, intellectual disability; HDACs, histone deacetylases; ncRNA, non-coding RNA.

Convergent node: chromatin remodeling at neuronal identity and synaptic enhancers. In ASD, mutations in CHD8, ARID1B, and ADNP silence genes critical for neuronal identity and synaptic scaffolding development. A specific chromatin-remodeling element in AD is less well characterized in current research than DNA and histone methylation pathways. This gap is noted here as a point of comparison rather than a confirmed convergence and does not imply a causal link from ASD to AD.

## 7 Direct molecular and genetic comparison: familial Alzheimer’s disease vs. monogenic autism

### 7.1 Familial Alzheimer’s disease: *APP*, *PSEN1*, *PSEN2* versus monogenic ASD syndromes

Familial AD (FAD) accounts for 5–10% of all AD cases, with pathogenic mutations identified in 25.5% of early-onset cases in a large French cohort of 5,496 patients, encompassing 90 distinct mutations and including 10 *de novo* PSEN1/APP mutations (Lanoiselée et al., 2017). Over 300 PSEN1 mutations have been documented globally, with PSEN mutations shifting γ-secretase cleavage to increase the Aβ42/Aβ40 ratio, which inversely correlates with age of onset, establishing a quantitative genotype–phenotype relationship between amyloidogenic cleavage bias and disease timing. A 2025 integrative multiomics study of iPSC-derived neurons from PSEN1, PSEN2, and APP mutation carriers identified common molecular endotypes—shared downstream cascades despite distinct primary mutations—across lipid metabolism, endolysosomal, and synaptic pathways (Valdes et al., 2025) (Tables 4 and 5).

**Table 4:** Condition-specific biomarkers in monogenic neurodevelopmental and neurodegenerative disorders.

Condition	Diagnostic biomarkers	Emerging research biomarkers	Therapeutic monitoring	References
Fragile X (FXS/FXTAS)	CGG repeat length; FMRP levels	mGluR5 PET; multiparametric EEG; executive function (FXTAS prodrome); digital gait/balance	Aβ40/42 in Fmr1-KO mice	Cabal-Herrera et al. (2020); Sadabad et al. (2024)
SHANK3/PMS	22q13 deletion/SHANK3 sequencing	CSF NfL/t-tau; computational MRI atrophy; EEG hyperconnectivity; SHANK3 expression; ERK activity	N/A	Jesse et al. (2023); Varella-Branco et al. (2026)
Familial AD	p-tau217, Aβ42/40 ratio	GFAP, NfL staging; Aβ profiles predicting age at onset; sTREM2, YKL-40; KP metabolites	p-tau217 as a trial endpoint; amyloid/tau PET	Palmqvist et al. (2025); Grande et al. (2025)

FMRP, fragile X mental retardation protein; MLPA, multiplex ligation-dependent probe amplification; MS-PCR, methylation-specific PCR; NfL, neurofilament light chain; ERK, extracellular signal-regulated kinase; KP, kynurenine pathway.

**Table 5:** Genetic and molecular comparison across familial Alzheimer’s disease and monogenic autism syndromes.

Feature	Familial AD	Fragile X	Prader-Willi	SHANK3/PMS
Genes	APP, PSEN1, PSEN2	FMR1 (CGG > 200)	15q11-q13 (SNORD116, MAGEL2)	SHANK3 (22q13.3)
Inheritance	AD (*de novo* 10%)	X-linked	Imprinting (paternal)	AD (de novo)
Protein function	γ-secretase; synaptotrophin; ER Ca^2+^	RNA-binding; translational repressor	snoRNA; ubiquitin ligase; necdin	PSD scaffolding; phase separation
Core mechanism	Aβ42/40↑; tau; ER Ca^2+^ leak; autophagy block	Protein synthesis↑; mGluR5-LTD; APP↑	H3K9me3 by G9a/SETDB1/ZNF274; Ih channelopathy	PSD condensate disruption
Onset	30–60 years	Infancy; FXTAS >50 years	Neonatal	Infancy; later regression
NDE connection	Primary NDE	FXTAS as NDE in premutation	Aging NDD phenotype; G9a overlap with AD	Progressive regression; AD gene overlap

AD, autosomal dominant; PSD, postsynaptic density; LTD, long-term depression; Ih, hyperpolarization-activated current; PMS, Phelan-McDermid syndrome; NDE, neurodegenerative disease.

FAD-PSEN2 mutants impair autophagy through a Ca^2+^-dependent, γ-secretase-independent mechanism: ER Ca^2+^ content reduction (~20%) impairs RAB7 recruitment to autophagosomes, blocking autophagosome-lysosome fusion. This shows that presenilin mutations have at least two independent pathogenic mechanisms—altered γ-secretase activity that produces toxic Aβ species and ER Ca^2+^ signaling disruption that impairs autophagy—both of which converge on proteinopathy and establish ER Ca^2+^ homeostasis as a non-amyloid therapeutic target in FAD (Fedeli et al., 2019).

This section’s evidence focuses on familial/monogenic AD and monogenic NDDs (FXS, PMS). As discussed in Section 7.3, findings from these monogenic subtypes—where a single genetic defect drives a specific molecular pathway—should not be broadly generalized to idiopathic ASD or sporadic late-onset AD, because these conditions involve more diverse, polygenic causes.

### 7.2 The APP–FMRP–mGluR5 axis as a molecular bridge

The APP–FMRP–mGluR5 triadic axis represents one of the most compelling molecular bridges linking the core biology of FXS with AD (Westmark and Malter, 2007; Spurrier et al., 2022; Sadabad et al., 2024). FMRP binds directly to APP mRNA at a G-quartet-like sequence in the coding region, constitutively repressing translation; upon mGluR5 activation—through DHPG or patterned synaptic input—this repression is lifted, and APP translation increases (Westmark and Malter, 2007). In Fmr1-KO synaptoneurosomes, basal APP translation is constitutively elevated, with increased soluble Aβ40 and Aβ42 across multiple mouse strains, demonstrating that FMRP loss creates a pro-amyloidogenic synaptic environment even in the absence of primary AD mutations.

The 2024 PET study showing 16–23% reduced mGluR5 availability in adults with ASD (Sadabad et al., 2024), together with the 2022 finding that the mGluR5 SAM BMS-984923 prevents C1q synaptic tagging and fully restores synapse density in aged AD mice (Spurrier et al., 2022), collectively establishes mGluR5 as a mechanistically justified convergent target. Therapeutic manipulation of mGluR5—whether through negative allosteric modulation (as in FXS trials) or silent allosteric modulation (as in the AD context)—may provide distinct but complementary benefits across the NDD-NDE spectrum. The fact that mGluR5 modulation simultaneously addresses APP translation dysregulation (relevant to FXS), synaptic density loss (relevant to AD), and complement-mediated pruning (relevant to both) makes this axis a paradigmatic example of how molecular convergence can guide transdiagnostic therapeutic development.

### 7.3 Idiopathic ASD as a complex and polygenic-epigenetic landscape

Idiopathic ASD, accounts for about 70–80% of cases without a specified monogenic cause, presents a distinct yet complementary challenge within the NDD–NDE continuum framework (Satterstrom et al., 2020; Gandal et al., 2022). Unlike FXS, PWS, or PMS, where a single genetic mutation drives a relatively predictable molecular cascade, idiopathic ASD is characterized by extreme genetic heterogeneity: numerous common variants of small effect, hundreds of rare *de novo* variants, and gene–environment interactions that converge on similar neurodevelopmental outcomes through multiple, partially overlapping pathological pathways (Satterstrom et al., 2020). This key difference shapes the genetic perspective of this review: evidence indicates that monogenic NDDs such as FXS and PMS have relatively strong mechanisms for NDD-NDE convergence, but these mechanisms cannot necessarily be generalized to idiopathic ASD. In idiopathic ASD, genetic convergence with AD may largely depend on whether the condition is familial or sporadic, a distinction examined throughout this section. Understanding how this molecular diversity produces shared clinical features and neurodegeneration risk is crucial for clarifying idiopathic ASD’s position within the NDD–NDE spectrum.

Whole-genome and whole-exome sequencing studies have identified more than 1,000 ASD-associated genes, yet the SFARI database’s highest-confidence (Category S and 1) risk genes—including *CHD8, SHANK3, DYRK1A, ADNP, ARID1B, SETD5, TBR1, KDM5C,* and *SYNGAP1*—converge primarily on three functional networks: chromatin remodeling and transcriptional regulation, synaptic scaffolding and glutamatergic signaling, and neuronal migration and cortical lamination (Satterstrom et al., 2020). A 2022 Science study profiling 725 brain samples across 11 regions from idiopathic ASD cases documented anterior-to-posterior transcriptomic dysregulation affecting 4,223 differentially expressed genes, with alternative splicing and isoform usage as major contributors. This pattern mirrors the cortical gradient of AD transcriptomic changes and suggests that idiopathic ASD and AD share a common topology of vulnerability despite different etiological origins (Gandal et al., 2022).

A central molecular signature of idiopathic ASD at the epigenetic level is disruption of the S-adenosylmethionine (SAM)-dependent methylation hierarchy. Genetic polymorphisms in folate-cycle enzymes (MTHFR C677T, FOLR1 G80A, RFC1 A80G) or autoimmune folate receptor blockade (FRAA, present in 71–75% of children with ASD) reduce SAM bioavailability, triggering a hierarchical prioritization in which Tier 1 housekeeping methyltransferases (DNMT1, PRMT1) maintain activity while Tier 3–4 developmental methyltransferases (G9a/EHMT2, PRMT8) show a 50–70% reduction (Bellver-Sanchis et al., 2024; Liu et al., 2025). This selective epigenetic vulnerability produces the characteristic “double lock” chromatin state: 15–25% genome-wide DNA hypomethylation combined with a 38% increase in repressive H3K9me2 and a 25% reduction in activating H3K4me2 in the prefrontal cortex, silencing genes encoding synaptic proteins (SHANK3, NRXN1, NLGN3), neurotransmitter receptors (NMDAR, GABA), and social behavior regulators (EGR1, OXTR, RELN) (Bellver-Sanchis et al., 2024; Liu et al., 2025).

The neuroinflammatory contribution to idiopathic ASD deserves particular emphasis, as it represents the principal pathway through which idiopathic ASD most closely resembles the pathophysiology of late-onset neurodegeneration. Postmortem transcriptomic studies of brains from individuals with idiopathic ASD reveal marked upregulation of microglial activation genes, complement components (C1q, C3, C4), and MHC class I molecules in the frontal and temporal cortices (Mottahedin et al., 2017). Notably, a 2026 cortical organoid study identified the master regulatory module M5—dysregulated in idiopathic ASD—as controlling neuroinflammatory gene networks through epigenetic mechanisms shared with AD (Gordon et al., 2026). The co-occurrence of microglial activation, complement overexpression, and H3K9me2 accumulation in idiopathic ASD creates a self-reinforcing cycle: neuroinflammatory cytokines transcriptionally activate G9a/EHMT2, increasing H3K9me2 at synaptic gene loci and further amplifying synaptic silencing and microglial reactivity (Mottahedin et al., 2017; Bellver-Sanchis et al., 2024).

From a longitudinal perspective, idiopathic ASD presents a distinct challenge for NDD–NDE continuum research: the absence of a single molecular anchor complicates biomarker stratification, yet the shared downstream pathology—reduced DTI–ALPS, elevated neuroinflammatory cytokines, KP dysregulation, and mitochondrial dysfunction—provides a set of convergent monitoring endpoints applicable across etiological subtypes (Launay et al., 2023; Khaliulin et al., 2025; Terrabuio et al., 2025). Epidemiological evidence of increased early-onset dementia in adults with ASD is relevant to this broader framework, but current population-based studies do not reliably distinguish idiopathic from syndromic ASD or Alzheimer’s disease from other dementia diagnoses (Vivanti et al., 2021). Consequently, the magnitude and nature of neurodegenerative risk specifically associated with idiopathic ASD remain uncertain and require dedicated longitudinal studies. Therapeutic implications include the observation that precision epigenetic interventions targeting the G9a/LSD1 axis, validated in ASD animal models and now showing activity in AD models, are rational candidates for trials enrolling biologically stratified adults with idiopathic ASD who have elevated NfL or GFAP as early neurodegeneration signals (Bellver-Sanchis et al., 2024).

### 7.4 Fragile X syndrome: FMR1

FXS, which has a prevalence of 1:4,000 males, results from CGG expansion (>200 repeats) that causes FMR1 promoter methylation and loss of FMRP, a multifunctional RNA-binding protein that regulate translation of hundreds of synaptic mRNAs (PSD-95, Arc, MAP1B, CaMKIIα, APP) through its KH2 and RGG RNA-binding domains. In striatal medium spiny neurons, FMRP’s KH2 and RGG domains are specifically required for normal PSD-95 elimination during synaptic maturation, and their disruption favors elimination of immature rather than mature spine types, fundamentally altering the structural architecture of the striatal connectome (Richter et al., 2015).

FMRP also regulates APP translation, providing a molecular connection between FXS-related translational dysregulation and pathways relevant to AD; the APP–FMRP–mGluR5 axis is discussed in detail in Section 7.2.

### 7.5 SHANK3-associated autism: Phelan-McDermid syndrome

A 2022 genotype–phenotype correlation study in a large PMS cohort found that deletion size and specific SHANK3 variant types significantly predict the severity and timing of regression. Patients with larger 22q13 deletions encompassing SHANK3 and neighboring genes tend to have earlier and more severe regression than those with isolated SHANK3 point mutations, suggesting that haploinsufficiency of neighboring genes modifies the core SHANK3 phenotype and accelerates the neurodegenerative trajectory (Levy et al., 2022).

The molecular consequences of SHANK3 haploinsufficiency for PSD organization and their potential relevance to age-related synaptic vulnerability are discussed in Section 3.1 (Jia et al., 2025; Varella-Branco et al., 2026).

### 7.6 ASD as heterochronic perturbation of neurodevelopment: a unifying framework

Earlier sections of this review have highlighted significant molecular, cellular, and epidemiological similarities between ASD and AD, focusing on shared disruptions in pathways such as synaptic structure, epigenetic regulation, neuroinflammation, and metabolism. The heterochrony hypothesis of ASD is also introduced as a unifying, testable—but not yet confirmed—theory that offers a developmental explanation for these parallels and suggests why ASD could be a risk factor for later neurodegeneration.

In developmental biology, heterochrony refers to alterations in the timing or rate of developmental processes relative to the ancestral or typical program (Crespi, 2013). In ASD, Crespi’s landmark analysis demonstrated that the core behavioral, cognitive, and neurobiological features of ASD systematically reflect the noncompletion or slowing of normal developmental trajectories rather than the emergence of qualitatively novel pathological states. Four domains illustrate this pattern clearly. First, restricted and repetitive behaviors—including insistence on sameness, ritualistic routines, and intense circumscribed interests—are characteristic of typically developing children aged 2–4 years and normally decline as prefrontal regulatory circuits mature; in ASD, these patterns persist beyond the expected developmental window. Second, the local processing bias documented in ASD—superior performance on embedded-figure tasks, enhanced pitch discrimination, and a detail-focused perceptual style—mirrors the perceptual strategy of young children before global integration develops and is consistent with a failure to complete the local-to-global transition in cortical processing hierarchies. Third, absolute pitch perception, retained in ASD at rates far exceeding those in the general population, reflects the preservation of an early developmental auditory processing mode that is typically overwritten by learned relative pitch. Fourth, social-communicative patterns in ASD, including reduced reciprocal social engagement and theory-of-mind difficulties, parallel the developmental stage of children before social cognition fully matures—a parallel that cannot be attributed to intellectual disability alone, as it persists in high-functioning individuals with ASD (Crespi, 2013).

The neurobiological substrate of this heterochrony model is directly supported by structural and functional imaging data reviewed elsewhere in this article. The default mode network (DMN), which underlies social cognition and self-referential processing, shows atypically slow maturation in ASD: DTI-ALPS reductions documented in children with ASD (Wang et al., 2025a) and diffusion tensor imaging studies consistently reveal a connectivity profile in ASD that resembles an earlier developmental stage—high short-range and low long-range connectivity—that normally transitions to the inverse pattern through late childhood and adolescence (Crespi, 2013; Tang et al., 2014). A 2025 resting-state fMRI study confirmed atypical maturation of the functional connectome hierarchy in ASD, with delayed cortical maturation in childhood followed by aberrantly accelerated development in adolescence and subsequent dysregulation in young adulthood (Lee et al., 2025). This nonlinear trajectory—delayed and then dysregulated rather than simply arrested—is incompatible with the view that ASD reflects global developmental arrest and is instead consistent with, though does not by itself prove, a model of heterochronic asynchrony: selected systems mature on atypical schedules, creating windows of vulnerability that could plausibly interact with age-related decline decades later.

At the molecular level, the most direct mechanistic substrate of heterochrony in ASD is impaired synaptic pruning. As reviewed in Sections 3.1 and 3.2, postmortem studies have documented excess synaptic density in ASD brains—particularly in layer V pyramidal neurons of the temporal cortex—attributable to mTOR hyperactivation, which suppresses the autophagy required for developmental synapse elimination (Tang et al., 2014). mTOR-mediated impairment of autophagy during critical developmental windows leaves an excessive number of immature synaptic contacts that would normally be eliminated, thereby preserving a highly connected, diffuse, short-range network architecture that functionally resembles an earlier developmental stage. The complement-mediated pruning axis reviewed in Section 4.2 provides a parallel mechanism: reduced microglial phagocytosis of C3-opsonized synapses, documented in several ASD mouse models, similarly results in insufficient pruning and retention of developmentally immature connectivity patterns (Stevens et al., 2007; Tang et al., 2014). The E/I imbalance documented in ASD—excess excitatory relative to inhibitory tone, reviewed in the context of mTOR and TREM2 pathways—is a direct electrophysiological correlate of this immature, overconnected synaptic state, which is also characteristic of early developmental brain states before GABA matures to its inhibitory function.

The heterochrony framework may help resolve an apparent paradox in the NDD-NDE relationship: why should conditions characterized by developmental slowing confer vulnerability to accelerated neurodegeneration? One candidate explanation proposed here, which require longitudinal validation, is that the same molecular machinery—mTOR signaling, autophagy flux, synaptic pruning by microglia, and glymphatic clearance—that fails to complete the developmental maturation program in ASD may also, under aging-related stress, fail to manage the proteostatic and homeostatic demands of the aging brain. A brain whose autophagy and microglial pruning mechanisms were compromised during development enters adulthood with preexisting deficits in cellular housekeeping that directly predispose to Aβ and tau accumulation when clearance demands increase with age (Tang et al., 2014). The elevated early-onset dementia risk in adults with ASD (Vivanti et al., 2021), the familial co-aggregation of autism and dementia (Chang et al., 2025), and the shared genetic architecture at the 2p23.3 locus affecting glymphatic function (Huang et al., 2025) are all consistent with this interpretation: developmental heterochrony is proposed to establish a biological vulnerability trajectory that may extend across the lifespan, although this sequential causal claim remains to be directly tested.

Critically, the heterochrony hypothesis is not equivalent to characterizing ASD as “global developmental delay”. Global developmental delay implies uniform slowing across all cognitive domains and motor functions, typically resolving or substantially improving over time; ASD, by contrast, is marked by domain-specific asynchrony—selective slowing in social-communicative systems, connectivity maturation, and sensory integration, while other capacities may be age-appropriate or even precocious (Katayama et al., 2016). This distinction has direct clinical implications: it predicts that standard developmental screening tools calibrated to global milestone acquisition will systematically mischaracterize the autistic brain’s developmental profile, and that cognitive assessments in aging adults with ASD must account for this atypical baseline when evaluating neurodegeneration risk. The divergence from global developmental delay is further supported by distinct genetic origins: while some genes predispose to ASD and others to global developmental delay, the overlap is partial, and the shared genes primarily encode regulators of developmental timing and synaptic maturation rather than growth factors that govern general neural proliferation (Satterstrom et al., 2020).

The heterochrony model also integrates naturally with the epigenetic framework central to this review. The selective deficit in Tier 3–4 developmental methyltransferases (G9a/EHMT2, PRMT8) described in Section 7.3 represents an epigenetic basis for heterochrony: if the methyltransferases responsible for establishing chromatin states associated with developmental progression are selectively compromised, the brain’s ability to complete normal maturational transitions may be impaired at the epigenomic level (Bellver-Sanchis et al., 2024). The resulting “epigenetic immaturity”—preserved H3K4me2 at developmental gene loci alongside reduced H3K9me2 at regulatory elements—could theoretically maintain transcriptional programs appropriate for earlier developmental stages, offering one candidate molecular mechanism for the behavioral and neurobiological heterochrony described above. G9a inhibitors, reviewed as cross-cutting epigenetic targets for both ASD (restoring synaptic gene expression) and AD (reducing Aβ and tau), could theoretically modulate this heterochronic epigenetic state, normalizing developmental progression in ASD while simultaneously reducing the downstream risk of protein aggregation associated with impaired epigenetic regulation during aging (Bellver-Sanchis et al., 2024).

The heterochrony hypothesis of ASD proposes that the disorder arises from specific timing differences in neurodevelopmental processes. Its primary mechanisms are thought to include disrupted mTOR–autophagy pathways that affect synaptic pruning, along with atypical connectivity development. This perspective complements the molecular overlaps between ASD and AD discussed in this article: these shared vulnerabilities may originate from developmental disturbances and aging-related effects on biological networks, although this remains a hypothesis rather than established causality. Viewing ASD as a heterochronic condition, rather than a fixed neurodevelopmental disorder, allows us to view the NDD–NDE spectrum as a continuum from delayed maturation to accelerated degeneration, potentially unified by common failures in cellular homeostasis across the lifespan.

## 8 Shared and distinct biomarkers across conditions

### 8.1 Fluid biomarkers (ATN-X framework in AD; emerging ASD panels)

The ATN (Amyloid/Tau/Neurodegeneration) biomarker framework provides biological disease staging for AD independent of clinical symptoms (Table 6). In Phase 3 trials, monoclonal antibodies such as aducanumab, lecanemab, and donanemab demonstrated substantial amyloid clearance and modest slowing of cognitive decline, confirming amyloid reduction as a disease-modifying mechanism (Zhang et al., 2026). In 2025, the Alzheimer’s Association released its first clinical practice guideline on blood-based biomarkers (BBMs), evaluating 31 BBM tests across 49 observational studies (Palmqvist et al., 2025). Plasma p-tau217 has emerged as the most promising single analyte, showing the highest diagnostic accuracy for AD among available blood-based markers (Palmqvist et al., 2025). In a community-based cohort of 2,000 Swedish adults (SNAC-K, 16 years of follow-up), p-tau217 and GFAP showed the strongest hazard ratios and predictive performance for incident AD dementia, underscoring their potential for presymptomatic surveillance (Grande et al., 2025).

**Table 6:** Biomarker comparison across Alzheimer’s disease and autism spectrum disorder.

Category	AD	ASD	Shared/Convergent	References
Amyloid	Plasma Aβ42/40; amyloid PET	sAPPα↑ in FXS; Aβ in Fmr1-KO	APP processing imbalance	Palmqvist et al. (2025); Westmark and Malter (2007)
Tau	p-tau217 (best single); p-Tau181, p-Tau231	CSF t-Tau in PMS regression	Tau marks NDD regression	Palmqvist et al. (2025); Jesse et al. (2023)
Neurodegeneration	NfL ≥ 40.09 pg./mL; GFAP ≥31.40 pg./mL	NfL in PMS; GFAP in acute NDD	NfL/GFAP for aging NDD monitoring	Fang et al. (2024); Jesse et al. (2023); He et al. (2021)
Metabolic	Ceramides; purines; KP metabolites	KYN/TRP ratio; kynurenate↓; purines; SCFAs	TRP-KP axis; purine dysfunction	Launay et al. (2023); Sorgdrager et al. (2019); Erb et al. (2019); Cieślak and Wojtczak (2018)
Oxidative	8-OHdG, isoprostanes, MDA	GSH/GSSG, isoprostanes, 4-HNE, SOD/GPx	Overlapping oxidative panels	Niu et al. (2015); Kuźniar-Pałka (2025)
Neuroimaging	Amyloid/tau PET; DTI-ALPS↓	DTI-ALPS↓; EEG; mGluR5 PET↓	DTI-ALPS as a candidate glymphatic imaging measure	Zhang Y. et al. (2025); Wang et al. (2025a); Sadabad et al. (2024)
Immune	sTREM2, YKL-40, complement (ATN-X)	IL-6, TNF-α, IL-1β, MCP-1; T cell subsets	Cytokine overlap; IFN/STING axis	Mottahedin et al. (2017); Jin et al. (2025); Liao et al. (2020); Heneka et al. (2025)

sAPPα, soluble APP alpha; NfL, neurofilament light chain; GFAP, glial fibrillary acidic protein; KP, kynurenine pathway; SCFAs, short-chain fatty acids; 8-OHdG, 8-hydroxy-2′-deoxyguanosine; MDA, malondialdehyde; GSH, glutathione; GSSG, glutathione disulfide; 4-HNE, 4-hydroxynonenal; SOD, superoxide dismutase; GPx, glutathione peroxidase; DTI-ALPS, diffusion tensor imaging along the perivascular space.

Serum NfL and GFAP serve as markers of neurodegeneration and astrocyte activation, respectively, with proposed diagnostic cutoffs of NfL ≥ 40.09 pg./mL and GFAP ≥31.40 pg./mL, yielding an AUC of 0.928 for AD diagnosis (Fang et al., 2024). The expanded AT(N)-X framework incorporates biomarkers of neuroinflammation, including sTREM2, YKL-40, and other glial markers, reflecting growing evidence that immune activation occurs early in the Alzheimer’s disease continuum and interacts bidirectionally with amyloid-β and tau pathology. Accordingly, inflammatory biomarkers are increasingly regarded as complementary to the classical AT(N) biomarkers for disease characterization and monitoring (Jack Jr et al., 2018; Benedet et al., 2021). A 2024 study reported that combining serum NfL, p-tau217, and cognitive tests can improve MCI prediction and provide new insights for AD treatment. This cross-sectional study included 157 community-dwelling adults aged 55 and older, divided into healthy control, MCI, and likely AD groups. Serum NfL, Aβ_42_, Aβ_40_, and p-tau217 showed significant specificity in predicting MCI, with Aβ_40_ outperforming the others (AUC = 0.707). The AUC for NfL was 0.646. Combining NfL with p-tau217 increased predictive accuracy (AUC = 0.687), while combining NfL with BNT further improved MCI prediction accuracy (AUC = 0.732). This combined NfL-plus-neuroinflammation strategy reflects the ATN-X framework’s growth evolution of amyloid and tau and provides a realistic multimarker template (Jing et al., 2024).

The parallel development of biomarker stratification strategies in ASD and AD, driven by recognition of biological heterogeneity within each diagnosis, reflects convergent precision medicine approaches to these distinct but biologically overlapping conditions.

### 8.2 Candidate cross-condition neuroimaging and neurodegeneration measures

DTI-ALPS has emerged as a candidate imaging measure related to glymphatic function and has been investigated independently in both ASD and AD cohorts. Reduced DTI-ALPS values have been reported in both conditions, although its diagnostic, prognostic, and longitudinal validity across NDD and NDE populations remains to be established (Wang et al., 2025a; Zhang Y. et al., 2025). mGluR5 PET with [^18^F]FPEB has demonstrated reduced mGluR5 availability at the group level in adults with ASD (Sadabad et al., 2024). These findings support further investigation of mGluR5 PET as a research measure of receptor availability and, potentially, target engagement; however, current evidence is insufficient to establish diagnostic or clinical monitoring utility.

EEG and other imaging measures may likewise provide complementary information on circuit-level dysfunction, but their cross-condition relevance remains exploratory (Varella-Branco et al., 2026). Together, these findings identify DTI-ALPS and mGluR5 PET as candidate imaging measures with potential relevance across neurodevelopmental and neurodegenerative research. Independent replication, longitudinal validation, and assessment of diagnostic and prognostic performance will be required before either can be considered a clinically applicable cross-condition biomarker.

NfL, GFAP, and total tau are increasingly examined in aging NDD populations as markers of active neurodegeneration, building on their established utility in AD (Jesse et al., 2023; Fang et al., 2024). In one study 504 older adults (median age 75 years) with memory problems were recruited from the Multidomain Alzheimer’s Preventive Trial (MAPT) (4-year follow-up) and classified as having mild cognitive impairment (MCI) or normal cognition (NC). Plasma NfL was measured, and cross-sectional and longitudinal associations were evaluated using mixed-effects linear models. Higher plasma NfL was associated with lower cognitive function in the overall population (MMSE: *β* = −0.007, 95% CI = −0.013 to −0.001; CCS: *β* = −0.003, 95% CI = −0.006 to −0.001). Among those with MCI, but not NC, higher plasma NfL was associated with lower CCS cross-sectionally (*β* = −0.003, 95% CI = −0.005 to −0.0002). The upper-quartile NfL group showed a greater decline in CCS over time under MCI conditions (*β* = −0.07, 95% CI = −0.12 to −0.01). These findings suggest that plasma NfL may predict cognitive impairment in patients with MCI (He et al., 2021).

Adults with PMS undergoing regression show elevated CSF NfL and tau with progressive brain atrophy, providing the first direct evidence that validated AD biomarkers can detect active neurodegeneration in NDD populations (Jesse et al., 2023). In psychiatric cohorts containing NDD individuals, elevated NfL is associated with autoimmune processes, elevated t-tau with catatonia, and elevated GFAP with acute- onset disorders, suggesting that these neurodegeneration markers carry distinct biological information even within NDD populations (Jesse et al., 2023). A critical implication of these findings is that NfL and GFAP monitoring in aging NDD cohorts could serve as early warning indicators for the onset of neurodegenerative processes, enabling intervention before clinical decline becomes apparent. A prospective biomarker surveillance study in adults with FXS, PMS, PWS, and other monogenic NDDs, tracking p-tau217, NfL, and GFAP longitudinally alongside cognitive assessments, would directly test whether developmental molecular vulnerabilities accelerate neurodegenerative biomarker trajectories.

## 9 Fundamental divergences

Despite the extensive molecular, cellular, and epidemiological convergences reviewed above, fundamental divergences between NDDs and NDEs remain, defining their distinct pathological identities and limiting the extent to which shared therapeutic strategies can be applied without modification (Lord et al., 2020; Wilson et al., 2023).

Biomarker maturity represents one key divergence: AD now has validated ATN clinical practice guidelines, plasma biomarker consensus criteria, and regulatory-approved PET tracers, whereas ASD biomarkers remain heterogeneous, lack consensus, and have no regulatory endorsement, reflecting the broader etiological heterogeneity of the spectrum (Palmqvist et al., 2025). The temporal trajectory differs fundamentally: NDDs manifest during critical developmental windows with lifelong persistence of the primary lesion, whereas NDEs emerge after decades of ostensibly normal function through progressive accumulation of proteinopathy and neuroinflammation (Lord et al., 2020; Wilson et al., 2023). The directionality of synaptic pathology also diverges: NDDs typically feature structural synaptic abnormalities from early development (altered spine density, connectivity, and PSD organization), whereas NDEs exhibit acquired synaptic loss driven by complement-mediated pruning, proteinopathy, and microglial phagocytosis (Carbonell et al., 2023; Ye et al., 2026).

An important distinction concerns the potential reversibility of specific molecular abnormalities and associated phenotypes. In preclinical ASD models, selected epigenetic alterations, including changes involving H3K9me2 and H3K4me2, can be modified pharmacologically in adulthood, with accompanying changes in molecular and behavioral phenotypes (Bellver-Sanchis et al., 2024). These findings indicate that some ASD-associated molecular vulnerabilities may remain pharmacologically responsive beyond early developmental periods, but they do not imply reversibility or disease modification of ASD itself. In contrast, established AD involves progressive and irreversible neuronal loss, which places different constraints on therapeutic recovery. Recognizing these distinctions is essential when considering whether molecular targets can be translated across neurodevelopmental and neurodegenerative conditions.

### 9.1 The NDD–NDE continuum as a multi-objective trade-off landscape: evolutionary constraints and molecular trade-offs

A powerful conceptual framework for understanding why NDDs and NDEs share so many molecular vulnerabilities—and why these vulnerabilities are difficult to fully resolve therapeutically—emerges from recent theoretical work in network neuroscience. Fakhar and Astle (2026) argue that the human brain is not optimally designed under any single objective; rather, it is the product of navigating a high-dimensional, multi-objective trade-off landscape shaped by evolutionary history and developmental constraints (Fakhar and Astle, 2026). It should be emphasized that this Pareto optimality framework is a theoretical, systems-level heuristic derived primarily from network-level modeling rather than from direct empirical demonstration at the molecular level; its application below to specific molecular pathways (mTOR–autophagy, complement pruning, G9a/EHMT2 methylation, wiring economy) is therefore offered as an illustrative interpretive lens rather than as an established mechanistic explanation. Under Pareto-optimality theory, no single brain configuration can simultaneously maximize all objectives: metabolic efficiency, communication speed, developmental plasticity, synaptic stability, and proteostatic resilience are fundamentally in tension. The brain occupies a position along a Pareto frontier, a set of solutions in which improving one objective necessarily compromises another. Crucially, both NDDs and NDEs can be reframed not as deviations from an ideal brain, but as atypical positions within this trade-off space—different weightings of competing biological objectives imposed by genetic, epigenetic, and environmental constraints across the lifespan.

This framework directly illuminates the molecular convergences reviewed in this article. The mTOR–autophagy axis (Section 3.2) exemplifies a fundamental trade-off between synaptic growth during development and proteostatic clearance in aging: mTOR hyperactivation during critical developmental windows in ASD maximizes synaptic connectivity and cortical expansion, conferring potential cognitive advantages in certain niches, but at the cost of suppressed autophagy, which reduces the brain’s capacity to clear misfolded proteins (Aβ, tau) across decades. This is not a simple pathological failure but a biological trade-off: the same mTOR activity that promotes neurodevelopmental exuberance later imposes proteostatic vulnerability. Similarly, complement-mediated synaptic pruning (Section 4.2) represents a trade-off between developmental circuit refinement and long-term synaptic maintenance. Insufficient complement-driven pruning in ASD preserves an immature, hyperconnected network state suited to certain perceptual and memory tasks (a developmental Pareto-optimal position), yet this same reduced pruning capacity renders the aging brain unable to eliminate pathologically tagged synapses, contributing to AD-associated synapse loss when the complement system is abnormally reactivated and C1q mRNA has been reported to increase by up to approximately 80-fold in vulnerable brain regions, including the hippocampus (Yasojima et al., 1999).

Epigenetic regulation offers perhaps the most molecularly explicit illustration of trade-off dynamics across the NDD–NDE continuum (Figure 3). G9a/EHMT2-mediated H3K9 methylation (Section 6.3) must balance two competing objectives: depositing repressive chromatin marks to silence developmental genes no longer required in mature neurons (supporting neuronal identity) and preserving transcriptional flexibility to permit adaptive gene expression in response to synaptic activity. In ASD with reduced SAM bioavailability, Tier 3–4 methyltransferases including G9a are selectively compromised—a prioritization that reflects the same metabolic cost–function trade-off identified at the network level by Fakhar and Astle (2026): housekeeping epigenetic functions (Tier 1–2) are preserved at the expense of developmental fine-tuning. Conversely, in AD, G9a upregulation and H3K9me2 accumulation reflect an overcompensatory attempt to silence stress-response and inflammatory genes, shifting the epigenetic landscape away from synaptic gene expression toward a survival-oriented but cognitively costly chromatin state (Bellver-Sanchis et al., 2024). The same enzymatic node thus sits at a molecular trade-off vertex, whose failure mode differs depending on developmental stage, cellular context, and direction of perturbation.

**Figure 3:**
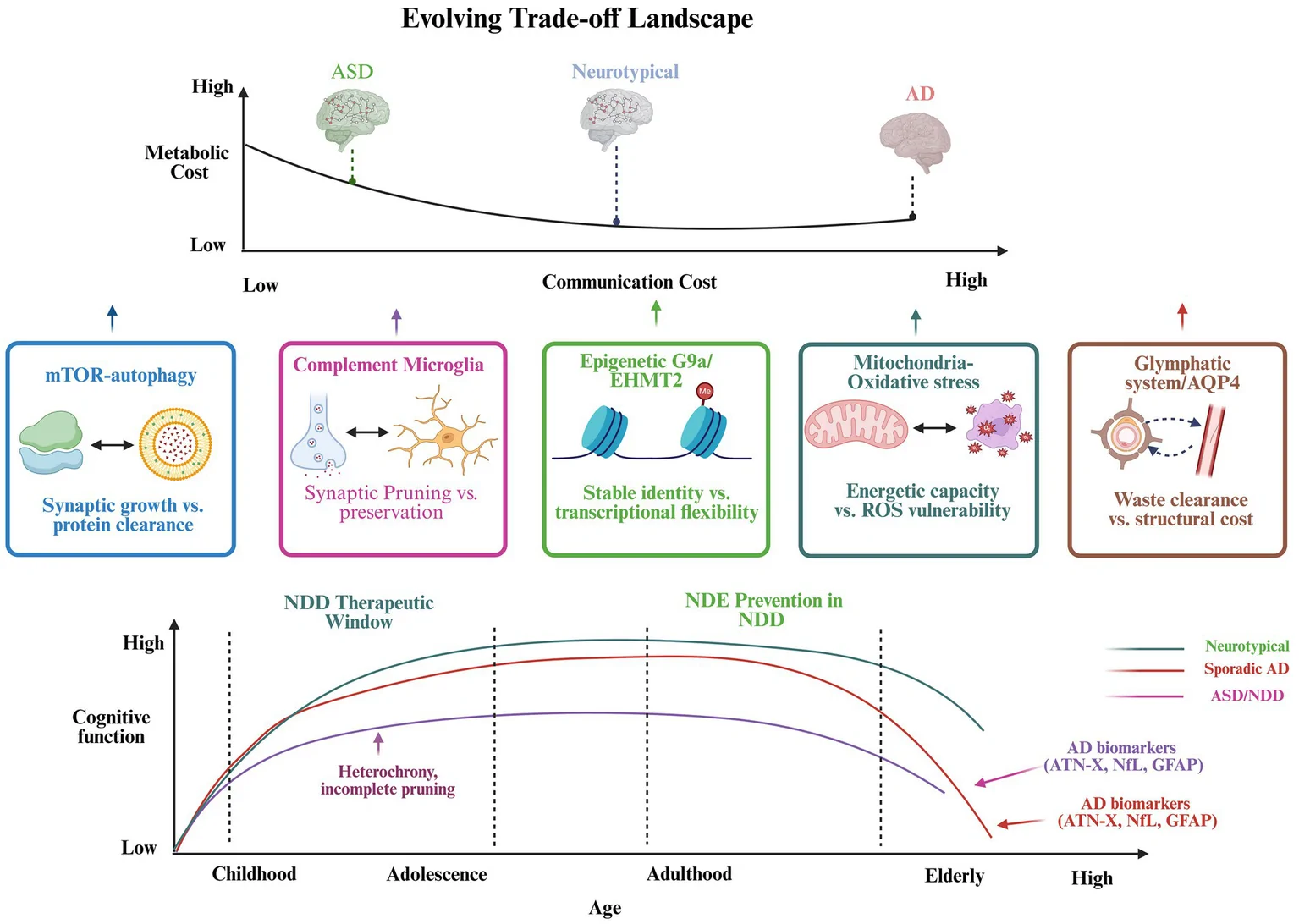
The NDD–NDE continuum as a trade-off landscape. Conceptual model in three stacked panels linking neurodevelopmental disorders (ASD) and neurodegenerative diseases (AD) as distinct positions within a shared landscape of evolutionary trade-offs. Top: Pareto diagram illustrating how the brain balances wiring and metabolic costs against communication efficiency, with relative positions for ASD, neurotypical, and AD configurations. Middle: Convergent molecular trade-off nodes (mTOR–autophagy, complement–microglia, G9a/epigenetics, mitochondria, glymphatic/AQP4) where these constraints are instantiated and shared vulnerabilities emerge. AD, Alzheimer’s disease; ASD, autism spectrum disorder; AQP4, aquaporin-4; APP, amyloid precursor protein; CR3, complement receptor 3; DTI-ALPS, diffusion tensor imaging along the perivascular space; NDD, neurodevelopmental disorder; NDE, neurodegenerative disease; ROS, reactive oxygen species.

The wiring economy–communication efficiency trade-off identified at the large-scale network level by Fakhar and Astle (2026) has a direct molecular parallel in the metabolic constraints shaping NDD and NDE vulnerability. Physical neural connections are metabolically costly, creating evolutionary pressure to minimize total wiring length while preserving interregional communication. The brain resolves this through small-world topology: a small set of expensive long-range connections (hubs) enables global communication at reduced total wiring cost. However, these metabolically expensive hubs are disproportionately vulnerable to both developmental disruption and age-related neurodegeneration. In ASD, the shift toward local over long-range connectivity—consistent with impaired synaptic pruning and heterochronic asynchrony (Section 7.6)—represents a reweighting toward lower wiring cost at the expense of communication efficiency, consistent with the Pareto- suboptimal interior of the trade-off landscape. In AD, the preferential degeneration of hippocampal-entorhinal hub connections and default mode network long-range projections represents the collapse of the same expensive-but-efficient architectural solution—a convergent failure at the high-cost end of the wiring trade-off. Mitochondrial dysfunction (Section 5.5), one of the most convergent features across the NDD–NDE spectrum, can be understood as the cellular-level expression of this same metabolic trade-off: the energetic demands of maintaining highly connected, metabolically expensive neurons exceed the sustainable output of mitochondrial complexes I–V when they are subject to oxidative stress, calcium overload, or epigenetic dysregulation.

The attractor-landscape perspective proposed by Fakhar and Astle (2026) provides additional explanatory power for the temporal dynamics of the NDD–NDE continuum. In dynamical systems theory, brain states are modeled as traversing a high-dimensional landscape of attractors—stable states toward which the system is drawn under perturbation. NDDs can be conceptualized as early developmental attractors: atypical but locally stable configurations of neural connectivity and molecular signaling reached through constrained developmental trajectories. These configurations are not random deviations from normality but Pareto-optimal solutions for a specific set of genetic, epigenetic, and environmental constraints experienced during early brain development. The NDE trajectory represents a forced transition from this developmental attractor toward a degenerative attractor, driven by the accumulation of proteotoxic stress, neuroinflammation, and metabolic exhaustion that the developmental configuration is ill-equipped to withstand. This formulation is consistent with the observation that ASD individuals face an increased risk of early-onset dementia (Vivanti et al., 2021); that the glymphatic system—responsible for waste clearance—shows shared genetic vulnerability across ASD children and AD patients at the 2p23.3 locus (Huang et al., 2025).

Understanding NDDs and NDEs through the lens of evolutionary trade-offs has direct therapeutic implications (Table 7). Fakhar and Astle emphasize that “atypical developmental trajectories do not represent mere deviations from a perfect, idealized brain; rather, they are reweighted compromises occupying the same high-dimensional space” (Fakhar and Astle, 2026). This perspective cautions against therapeutic strategies that seek to “normalize” a single molecular parameter without accounting for the trade-offs embedded in the broader molecular network. The opposing effects of mTOR inhibition in neurons (beneficial: reduced tau aggregation) versus microglia (detrimental: suppressed TREM2-dependent Aβ clearance) (Section 3.2) are a direct molecular manifestation of this trade-off complexity: there is no globally optimal mTOR activity level, only Pareto-optimal solutions that weight neuronal proteostasis against microglial immune function differently. G9a inhibition illustrates the same principle: normalizing H3K9me2 in ASD restores synaptic gene expression and social behavior (Bellver-Sanchis et al., 2024), but excessive reduction of H3K9me2 in AD neurons may derepress retrotransposons and inflammatory programs—a trade-off that requires cell-type-selective, dose-titrated therapeutic delivery. Mapping the molecular trade-off landscape of the NDD–NDE continuum—identifying which biological objectives are in tension at each convergent node—thus becomes a prerequisite for rational transdiagnostic therapeutic design, guiding the development of interventions that shift patients toward better trade-off positions rather than simply optimizing a single molecular endpoint.

**Table 7:** Evolutionary trade-offs and convergent molecular pathways across the NDD–NDE continuum.

Trade-off	Molecular pathway	Relevance across the NDD–NDE continuum	References
Synaptic growth vs. proteostasis	mTOR–autophagy	mTOR hyperactivation in ASD promotes synaptic overgrowth and reduced pruning, whereas chronic mTOR activation in AD increases APP/tau burden and suppresses autophagic clearance.	Lipton and Sahin (2014); Xie et al. (2024); Trifonova et al. (2025)
Circuit refinement vs. synaptic preservation	Complement–microglia (C1q/C3/CR3)	Reduced or altered pruning in ASD contributes to immature hyperconnected networks, while complement reactivation in AD drives early synapse loss and microglial phagocytosis.	Spurrier et al. (2022); Stevens et al. (2007); Ye et al. (2026)
Stable neuronal identity vs. transcriptional flexibility	G9a/EHMT2-mediated H3K9 methylation	In ASD, developmental methyltransferase vulnerability may impair maturational gene silencing; in AD, G9a upregulation and H3K9me2 accumulation repress synaptic programs and favor stress-related states.	Karwacki-Neisius et al. (2024); Bellver-Sanchis et al. (2025)
Wiring economy vs. communication efficiency	Small-world network organization/hub vulnerability	ASD tends to shift toward local rather than long-range connectivity, whereas AD preferentially affects metabolically costly long-range hubs, linking connectome architecture to degeneration risk.	Lee et al. (2025); Fakhar and Astle (2026)
Energetic capacity vs. oxidative vulnerability	Mitochondria–ROS/mitophagy	Shared mitochondrial dysfunction across ASD and AD reflects the energetic burden of maintaining highly connected neurons under oxidative, calcium, and proteostatic stress.	Paulsen et al. (2022); Khaliulin et al. (2025); D’Alessandro et al. (2025)
Waste clearance vs. developmental/structural constraints	Glymphatic system/AQP4/DTI-ALPS	The glymphatic reduction observed in ASD and AD suggests a potential shared vulnerability, whose longitudinal significance remains to be established.	Zhang Y. et al. (2025); Wang et al. (2025a); Dagum et al. (2026); Huang et al. (2025)
Developmental attractor stability vs. aging resilience	Attractor landscape/heterochrony framework	ASD can be viewed as an early, locally stable developmental attractor that becomes increasingly vulnerable to proteotoxic, inflammatory, and metabolic stress with aging.	Fakhar and Astle (2026); Crespi (2013); Lee et al. (2025)

## 10 Alzheimer’s disease vs. autism spectrum disorder: direct molecular comparison

A phylostratigraphic analysis conducted in 2025 showed that genes associated with both ASD and AD are predominantly ancient. ASD genes, in particular, exhibit signs of stabilizing selection, suggesting that genetic variants related to autism are ancient regulatory elements that are highly sensitive to developmental disturbances. The 148 shared genes and hub nodes (including CTNNB1, CREBBP, TRAF2, HDAC1, and HDAC2) identified at the intersection of ASD and AD form a core set of developmental-regulatory proteins. Disruptions in their expression during development lead to neurodevelopmental disorder (NDD) features, while similar disruptions during aging result in neurodegenerative disorder (NDE) features(Trifonova et al., 2025), as illustrated in Figure 4.

**Figure 4:**
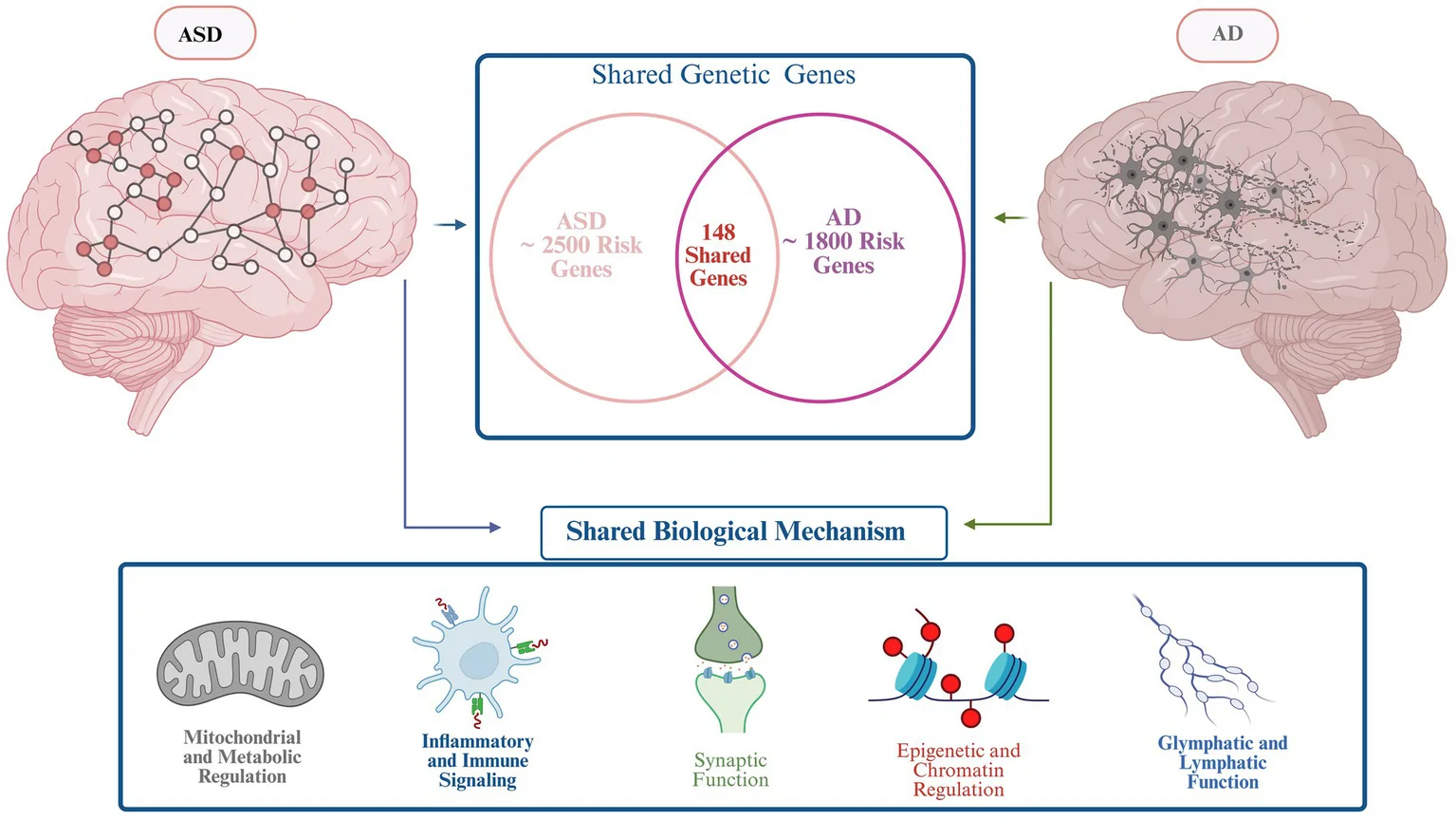
Alzheimer’s disease vs. autism spectrum disorder.

In 2022, a pioneering study employed a Mendelian randomization framework to examine potential causal links between genetic vulnerability to ADHD, ASD, and Alzheimer’s disease, using the largest publicly available sample sizes for each condition. The study analyzed the most extensive GWAS datasets of European ancestry, which included 20,183 ADHD cases with 35,191 controls, 18,381 ASD cases with 27,969 controls, 71,880 Alzheimer’s cases with 383,378 controls, as well as GWAS data for educational attainment (766,345 participants) and IQ (269,867 participants). The findings indicated that genetic susceptibility to Alzheimer’s did not significantly increase the risk of ADHD or ASD (OR = 1.12, 95% CI: 0.86–1.44, *p* = 0.37 for ADHD; OR = 1.19, 95% CI: 0.94–1.51, *p* = 0.14 for ASD), providing limited evidence for a direct causal relationship (Pagoni et al., 2022). This null Mendelian randomization finding is an important caveat for the overall NDD–NDE continuum framework, as it indicates that genetic liability to AD does not causally predispose individuals to ASD. The convergences described throughout this review should therefore be understood as shared downstream biological vulnerabilities rather than direct bidirectional genetic causation.

Glymphatic dysfunction represents another potential point of convergence between ASD and AD. Cross-sectional imaging and genetic findings relevant to this hypothesis are reviewed in Section 5.4, but they do not establish a longitudinal developmental-to-degenerative pathway. These observations may be interpreted within the heterochrony framework proposed in Section 7.6, although prospective studies are required to determine whether early alterations in homeostatic or clearance mechanisms influence later neurodegenerative vulnerability.

## 11 Therapeutic convergence: epigenetic and multi-target strategies

The extensive molecular convergences between NDDs and NDEs reviewed above provide a principled basis for transdiagnostic therapeutic strategies. Rather than developing entirely separate intervention pipelines for each condition, the shared biological nodes identified in this review offer opportunities for rational repurposing and cross-condition development (Lipton and Sahin, 2014; Spurrier et al., 2022; Bellver-Sanchis et al., 2024; Xie et al., 2024).

G9a inhibitors are among the most well-validated epigenetic targets across conditions. A brain-penetrant G9a methylase inhibitor has been shown to reduce both Aβ and tau pathology in Alzheimer’s disease (AD) mouse models (Xie et al., 2025). In autism spectrum disorder (ASD) models, particularly Shank3-deficient mice, G9a inhibition reduces H3K9me2 accumulation, restores synaptic gene expression, and improves social behavior (Wang et al., 2020). A 2025 article in Medicinal Research Reviews highlights G9a inhibitors as a leading epigenetic approach for various neuropsychiatric disorders within the NDD-NDE spectrum (Bellver-Sanchis et al., 2025). Additionally, HDAC modulators—particularly HDAC2 inhibitors for memory improvement in AD and HDAC3 inhibitors for extinction memory in NDDs—are promising pharmacological targets supported by preclinical evidence (Guan et al., 2009).

mTOR inhibition presents a more complex therapeutic landscape because of opposing effects in neurons versus microglia: rapamycin suppresses beneficial TREM2-dependent Aβ clearance in microglia while reducing tau aggregation in neurons (Morawe et al., 2022; Shi et al., 2022). A single-site, open-label phase 1 clinical trial investigating the effects of rapamycin in 10 participants (mean age 74 ± 4 years, 60% female) provided first-in-human evidence that systemic mTOR inhibition has pharmacological effects on both ASD and AD molecular targets (Gonzales et al., 2025). This cellular specificity mandates targeted delivery approaches—potentially using cell-type-selective nanoparticles or prodrug strategies—to exploit convergent mTOR vulnerability without compromising protective microglial function (Xie et al., 2024; Trifonova et al., 2025). mGluR5 modulation, complement inhibition targeting C1q/C3, and microbiome-directed interventions complete a mechanistically justified transdiagnostic therapeutic portfolio(Stevens et al., 2007; Spurrier et al., 2022; Yan et al., 2025).

Therapeutic strategies targeting convergent molecular pathways must also account for developmental timing. Preclinical ASD models indicate that selected epigenetic abnormalities and associated phenotypes can remain pharmacologically modifiable in adulthood (Wang et al., 2020; Bellver-Sanchis et al., 2024). This finding supports further investigation of adult interventions directed at defined molecular vulnerabilities, but it does not establish disease-modifying effects on ASD itself. Epigenetic targets such as G9a/EHMT2 and LSD1/KDM1A may therefore warrant longitudinal study using molecular, behavioral, and neurodegeneration-related outcomes to determine the extent and clinical relevance of such modulation.

## 12 Future directions and open questions

The central priority for future research is to determine whether the molecular and physiological overlaps described in this review have longitudinal relevance. First, prospective studies of aging individuals with ASD and other NDDs should determine whether trajectories of established or clinically advanced neurodegeneration biomarkers, including p-tau217, NfL, and GFAP, differ from those observed in appropriately matched comparison populations (Fang et al., 2024; Grande et al., 2025; Palmqvist et al., 2025). Evidence from Phelan–McDermid syndrome showing increased NfL and tau in individuals undergoing neurological regression further supports the feasibility of investigating neurodegeneration-related biomarkers longitudinally in selected NDD populations, although these markers are not yet validated for this purpose across NDDs (Jesse et al., 2023). Candidate imaging measures such as DTI-ALPS could be incorporated alongside cognitive and neurological assessments, while remaining clearly distinguished from clinically established AD biomarkers (Wang et al., 2025a; Zhang Y. et al., 2025).

Second, it will be important to determine whether the glymphatic alterations discussed in Section 5.4 have any relationship with subsequent neurodegenerative vulnerability. Reduced DTI-ALPS values have been reported independently in ASD and AD populations, but current cross-sectional findings do not establish a longitudinal relationship between early-life alterations in clearance-related processes and later neurodegeneration (Wang et al., 2025a; Zhang Y. et al., 2025). Prospective studies combining glymphatic-related imaging with established AD biomarkers could therefore test whether early alterations in clearance-related measures predict subsequent changes in Aβ, tau, or other markers of neurodegeneration. Sleep-dependent changes in the clearance of brain-derived AD biomarkers provide an additional rationale for investigating sleep and other potentially modifiable physiological influences on glymphatic function longitudinally (Dagum et al., 2026).

Third, the heterochrony framework proposed in Section 7.6 should be tested directly rather than inferred from cross-sectional similarities. Longitudinal neuroimaging and molecular studies could determine whether atypical developmental trajectories of functional connectivity, synaptic pruning, or related homeostatic systems are associated with subsequent changes in neurodegeneration biomarkers (Crespi, 2013; Tang et al., 2014; Lee et al., 2025). Similarly, preclinical evidence that selected epigenetic abnormalities and associated behavioral or synaptic phenotypes remain pharmacologically modifiable in adulthood supports further investigation of defined molecular vulnerabilities, without implying reversibility or disease modification of ASD itself (Wang et al., 2020).

In summary, ASD and AD share several molecular and cellular pathways, but convergence of biological mechanisms does not demonstrate that ASD progresses to AD or that early neurodevelopmental abnormalities causally produce later neurodegeneration. Indeed, genetic evidence has not established a direct causal relationship between liability to AD and ASD (Pagoni et al., 2022). The principal testable question is therefore whether specific developmental vulnerabilities influence longitudinal trajectories of established neurodegeneration biomarkers during aging. Addressing this question will require prospective, well-phenotyped cohorts and a clear distinction between shared biological pathways, candidate biomarkers, predictive associations, and causal mechanisms.
